# Discovery of 4,6-disubstituted pyrimidines as potent inhibitors of the heat shock factor 1 (HSF1) stress pathway and CDK9†
†The authors declare the following competing financial interest(s): C. Rye, N. Chessum, L. Zani, M. Cheeseman, F. Raynaud, A. Hayes, A. Henley, E. de Billy, C. Lynch, S. Sharp, R. te Poele, L. O'Fee, P. Workman, and K. Jones are or have been employees of The Institute of Cancer Research which has a commercial interest in the development of HSF1 inhibitors. Authors who are or have been employed by The Institute of Cancer Research are subject to a *“*Rewards to Discoverers Scheme*”* which may reward contributors to a program that is subsequently licensed.
‡
‡Electronic supplementary information (ESI) available: Experimental and assay procedures, crystallographic data, compound characterisation data. See DOI: 10.1039/c6md00159a



**DOI:** 10.1039/c6md00159a

**Published:** 2016-06-13

**Authors:** Carl S. Rye, Nicola E. A. Chessum, Scott Lamont, Kurt G. Pike, Paul Faulder, Julie Demeritt, Paul Kemmitt, Julie Tucker, Lorenzo Zani, Matthew D. Cheeseman, Rosie Isaac, Louise Goodwin, Joanna Boros, Florence Raynaud, Angela Hayes, Alan T. Henley, Emmanuel de Billy, Christopher J. Lynch, Swee Y. Sharp, Robert te Poele, Lisa O’ Fee, Kevin M. Foote, Stephen Green, Paul Workman, Keith Jones

**Affiliations:** a Cancer Research UK Cancer Therapeutics Unit , The Institute of Cancer Research , London SW7 3RP , UK . Email: Paul.Workman@icr.ac.uk ; Email: Keith.Jones@icr.ac.uk; b AstraZeneca , Alderley Park , Macclesfield , Cheshire , SK10 4TG , UK

## Abstract

Heat shock factor 1 (HSF1) is a transcription factor that plays key roles in cancer, including providing a mechanism for cell survival under proteotoxic stress. Therefore, inhibition of the HSF1-stress pathway represents an exciting new opportunity in cancer treatment. We employed an unbiased phenotypic screen to discover inhibitors of the HSF1-stress pathway. Using this approach we identified an initial hit (**1**) based on a 4,6-pyrimidine scaffold (2.00 μM). Optimisation of cellular SAR led to an inhibitor with improved potency (**25**, 15 nM) in the HSF1 phenotypic assay. The 4,6-pyrimidine **25** was also shown to have high potency against the CDK9 enzyme (3 nM).

## Abstract 2

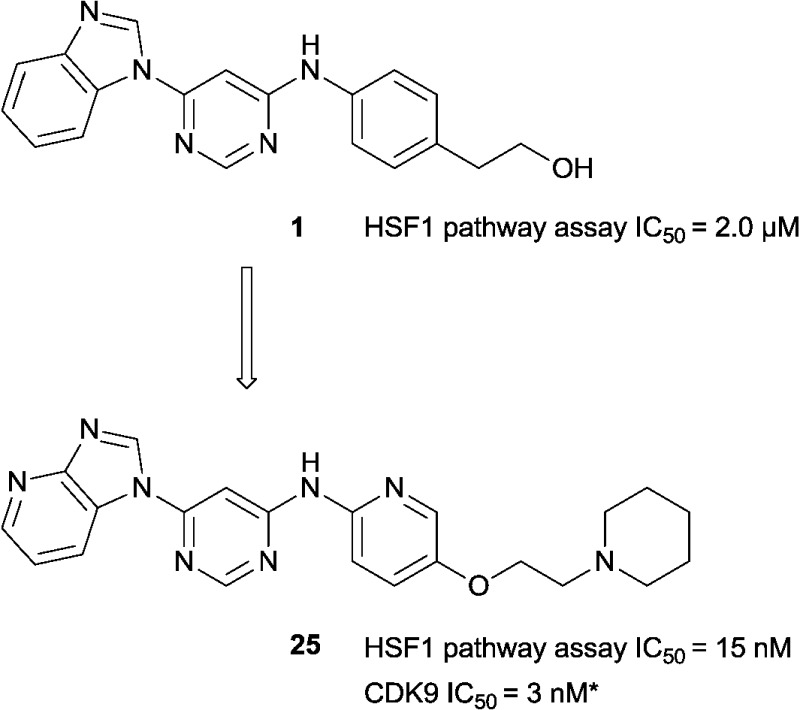
A series of 4,6-disubstituted pyrimidines from a phenotypic screen targeting the HSF1 pathway are also potent inhibitors of CDK9.

## 1. Introduction

Heat shock factor 1 (HSF1) is a transcription factor that is the master regulator of the canonical heat shock response, modulating the expression of hundreds of genes critical to the survival of the cell.^1–3^ HSF1 is implicated in the cellular response to a variety of stressors and plays a key role in oncogenesis and malignant progression, among other benefits enabling the cell to cope with the proteotoxic stress resulting from malignant transformation.^4,5^

In addition to its transient activation in the classical heat shock response, HSF1 is frequently upregulated in human cancers.^4,6–8^ An HSF1-regulated transcriptional program has been identified that is specific to highly malignant cells, overlapping with but distinct from the heat shock response, which is strongly associated with metastasis and poor survival in cancer patients.^9^ There are multiple mechanisms by which HSF1 has been proposed to facilitate oncogenesis. HSF1 upregulates proteins involved in diverse biological processes which include cell cycle progression, survival, glucose metabolism, DNA repair and chromatin re-modelling.^4,10^ Furthermore, HSF1 supports malignant progression by promoting tumour invasion, angiogenesis and metastasis,^11–13^ which includes the re-programming of stromal cells within the tumour microenvironment.^14^

A key feature in the HSF1-mediated response to proteotoxic stress is the upregulation of heat shock proteins (HSPs) including HSP72 and HSP90.^15^ The HSPs are chaperone proteins critical for proper protein folding, preventing self-association, maintaining active multi-protein complexes and directing misfolded proteins to be degraded.^16,17^ In addition, depletion of HSF1 destabilizes ribosomal subunit proteins, which reveals a link between cellular chaperoning and translational capacity.^18^ Importantly there is a positive correlation between increased expression of nuclear (activated) HSF1 and HSPs and poor patient outcome, including poor prognosis in many breast cancers.^6,9^

Taken together, the above results support the exciting possibility that inhibiting the HSF1-stress pathway could represent a novel therapeutic strategy that would deliver strong selective effects against cancer cells. This is supported by target validation studies using knockdown of HSF1 by genetic means.^4,19^

A number of structurally diverse compounds have been reported to act as inhibitors of HSF1 or the HSF1-stress pathway, *via* a variety of proposed mechanisms of action.^8,20^ However, HSF1 is a ligand-less transcription factor with poor predicted druggability and as such is difficult to inhibit directly using a small molecule approach. Consequently, we decided to conduct an unbiased cell-based phenotypic screen to identify inhibitors of the HSF1-stress pathway.

## 2. Results and discussion

### 2.1. Hit identification

To discover inhibitors of the HSF1-stress pathway, we employed an automated cellular imaging and analysis method (ArrayScan™) that quantifies the ability of a compound to suppress the expression of the HSF1-mediated inducible HSP70 isoform, HSP72. Cancer cells were treated with 17-allylamino-17-demethyoxygeldanamycin (17-AAG) an HSP90 inhibitor known to stimulate an HSF1-mediated response^21,22^ and compounds that blocked expression of HSP72 were thereby defined as inhibitors of the HSF1-stress pathway.

**Fig. 1:**
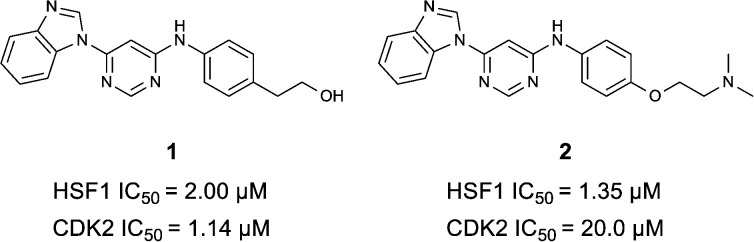
High-throughput screening hit pyrimidine **1** and dimethylamino-containing analogue **2**.

Approximately 200 000 small molecules (consisting of 35 000 kinase-directed compounds and a diversity set of 165 000 compounds from the AstraZeneca collection) were screened using this approach in the U2OS human osteosarcoma tumour cell line. One of the hits selected for progression was the 4,6-disubstituted pyrimidine **1** which, following re-synthesis, was confirmed as active with a cellular IC_50_ value of 2.00 μM for HSF1-stress pathway inhibition (Fig. 1).

In-house data revealed that 4,6-pyrimidine **1** also possessed modest CDK2 activity with an IC_50_ value of 1.14 μM in a biochemical assay, though it was unclear at this stage whether this kinase activity was important for the observed HSF1 cellular phenotype.

**Fig. 2:**
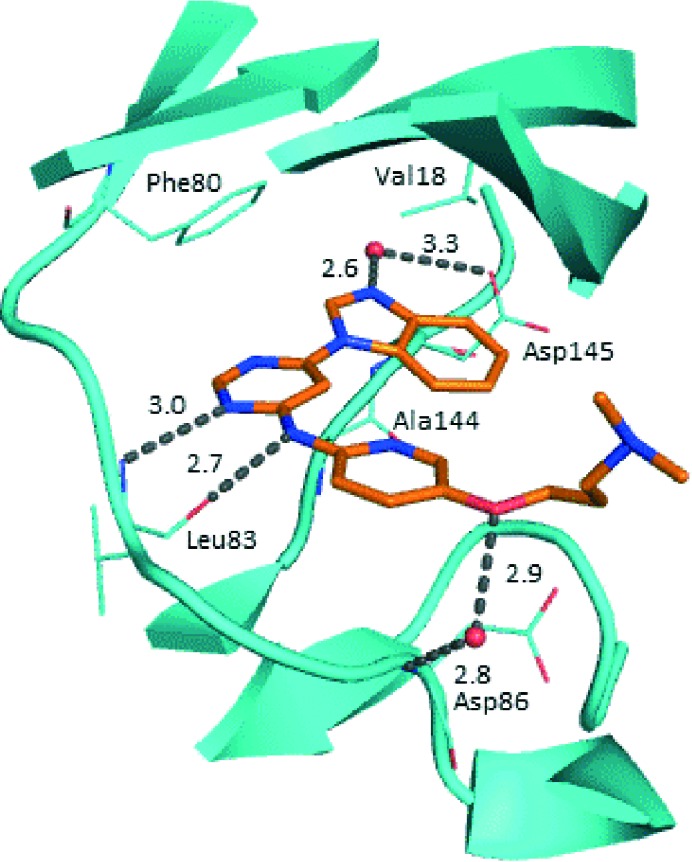
X-ray co-crystal structure of **4** bound to CDK2. Measurements in Å. PDB ID ; 4BZD.

Prior to investigating the structure activity relationship (SAR) it was necessary to improve the solubility of alcohol **1**. To achieve this, the phenethyl alcohol chain was replaced with an oxygen-linked dimethylamino side chain to give **2**. This modification retained potency in the HSF1-stress pathway assay (1.35 μM), but was less potent against CDK2 (20.0 μM). Preliminary explorations of the SAR (Table 1) were initiated to assess the effect that structural changes would have on both the HSF1-stress pathway activity and biochemical CDK2 activity, using the dimethylamino-containing compound **2** as a starting point. Substitution of the phenyl ring for a 2-pyridine ring (**3**) afforded a compound which was approximately 15-fold more potent in the HSF1-stress pathway assay and 35-fold more active against CDK2 when compared with phenyl compound **2**. To facilitate progression of this series we attempted to obtain a crystal structure of 2-pyridine **3** bound to CDK2, but without success. Fortunately, adding an extra methylene to the side chain (**4**) afforded an analogue with a modest improvement in affinity for CDK2 which could be used to generate a crystal structure (Fig. 2). The CDK2 used for crystallisation was expressed, purified and crystallised in the absence of cyclin. Analysis of the crystal structure revealed that the pyrimidine N1 and 6-NH group form a typical hydrogen bond pair interaction with Leu83 (ref. 23) in the hinge region of CDK2. The benzimidazole N3 is observed to form a water-mediated hydrogen bond to the side-chain of Asp145 and the ether oxygen is involved in a water-mediated hydrogen bond to Asp86. The benzimidazole ring system stacks between three lipophilic residues Phe80, Val18 and Ala144.

**Table 1:** Investigation of SAR surrounding 4,6-pyrimidines for HSF1-stress pathway and CDK2 assays

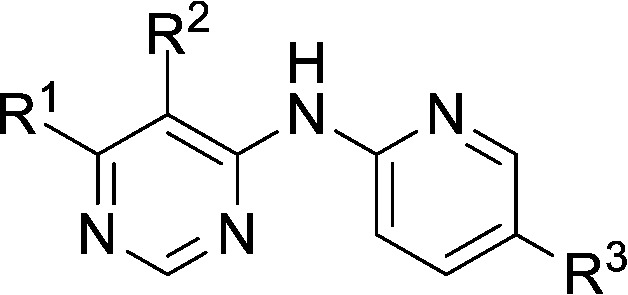 general structure for Table 1					
Compound	R^1^	R^2^	R^3^	HSF1 IC_50_ ^*a*^ (μM)	CDK2 IC_50_ ^*a*^ (μM)
**3**	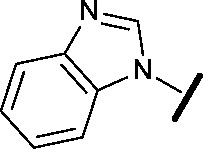	H	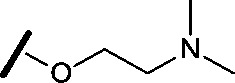	0.080	0.580
**4**	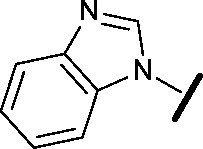	H	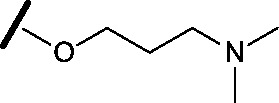	0.165	0.368
**5**	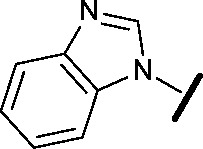	Me	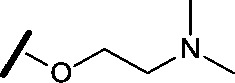	>30	65.8
**6**	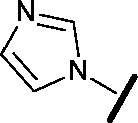	H	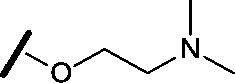	6.15	7.10
**7**	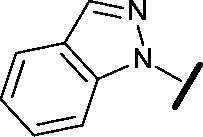	H	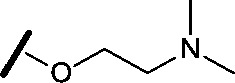	>30	29.8
**8**	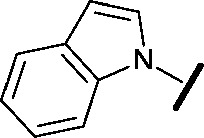	H	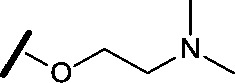	2.33	4.43
**9**	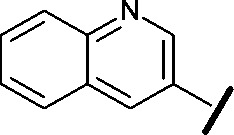	H	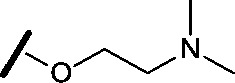	17.6	>100
**10**	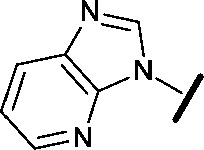	H	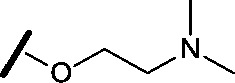	0.760	1.60
**11**	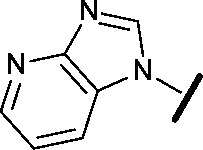	H	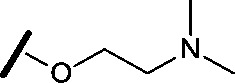	0.028	0.161
**12**	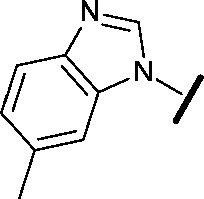	H	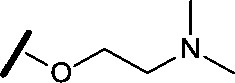	0.039	0.121
**13**	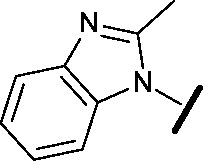	H	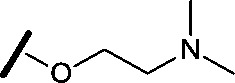	4.28	4.61
**14**	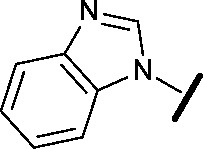	H	H	0.842	0.059
**15**	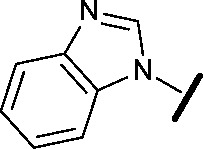	H	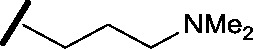	0.198	0.690
**16**	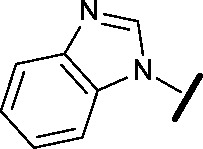	H	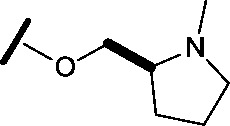	0.055	0.535
**17**	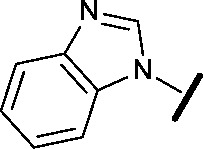	H	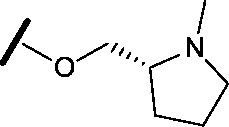	0.045	0.492
**18**	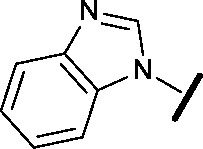	H	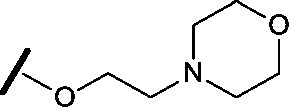	0.295	0.554
**19**	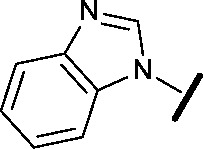	H	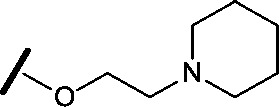	0.057	0.358
**20**	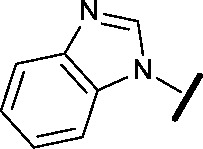	H	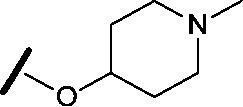	0.884	1.24
**21**	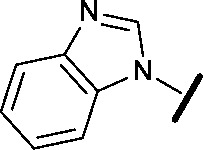	H	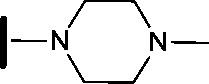	0.450	0.643
**22**	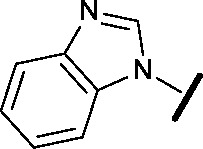	H	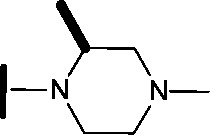	1.38	0.836
**23**	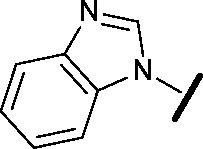	H	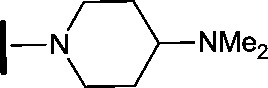	0.610	0.776

^*a*^Potency data are reported as the average of two determinations.

With the crystal structure in hand, we were in a position to explore the hypothesis that inhibition of CDK2 forms the basis of the observed HSF1-stress pathway inhibition. Using single structural changes with respect to 4,6-disubstituted pyrimidine **3** and comparing the activity of the compounds generated we aimed to distinguish which features of the SAR gave potency and selectivity for each assay.

### 2.2. Rational analogue design

We began by examining the role of the central pyrimidine ring. Introduction of a methyl group at C-5 to afford pyrimidine (**5**) was predicted to cause a steric clash with the other substituents on the ring, disfavouring the planar binding conformation and disrupting CDK2 binding. In both the CDK2 and the HSF1-stress pathway assays a large drop in activity was measured (>100- and >300-fold respectively). Next, we examined the contribution of the benzimidazole head group. Compounds were designed that would interrogate whether the water-mediated hydrogen bond to Asp145, and the ability of the group to be involved in π-stacking, were important for CDK2 activity and whether this would also impact the HSF1-stress pathway activity.

Exploration commenced with imidazole **6**, a truncated compound that is able to form the required hydrogen bond, but cannot be involved in π-stacking. A drop in potency was observed with imidazole **6** in the HSF1-stress pathway assay (77-fold) and to a lesser extent the CDK2 biochemical assay (12-fold). Indazole **7** and indole **8** are unable to form a hydrogen bond with Asp145 and showed a drop in potency in both assays. The corresponding quinoline **9** is capable of forming a weak hydrogen bond whilst maintaining the ability for π-stacking. However, this compound was also less potent (∼200 fold for the HSF1-stress pathway assay and inactive in the CDK2 assay) as compared to benzimidazole **3**. 7-Azabenzimidazole 10 has the potential for H-bond formation, but a polar group has been introduced onto the aromatic ring and a loss in potency is observed in both assays compared to benzimidazole **3**. Surprisingly the introduction of a nitrogen atom at C-4 of the benzimidazole ring, to afford 4-azabenzimidazole **11** led to a 3-fold increase in HSF1-stress pathway inhibition and a 5-fold improvement in CDK2 inhibition compared with benzimidazole **3**. Finally, the co-crystal structure of **4** with CDK2 suggested a small lipophilic substituent at C-6 of the benzimidazole could be accommodated. This hypothesis was confirmed with 6-methylbenzimidazole **12** and which showed a small increase in potency in both assays. In contrast, substitution at C-2 of the benzimidazole was predicted to create a steric clash with the phenylalanine gatekeeper of CDK2. This hypothesis was confirmed with 2-methylbenzimidazole **13**, where a >7-fold loss in activity was observed in both assays. In summary, modifications to the benzimidazole ring showed that the 4-azabenzimidazole was the most potent substituent at this position.

In the CDK2 co-crystal structure the amine tail resides in a solvent channel. Cho *et al.* have demonstrated that bulky amine groups at the periphery of the ATP binding site clash with Lys89 of CDK2 and help improve selectivity towards other kinase family members, such as CDK4 and CDK6.^24^ Therefore, in an attempt to examine whether CDK2 and HSF1-stress pathway inhibition correlated, several analogues were synthesised to explore the impact of variation at this vector on CDK2 and HSF1-stress pathway activity.

Removal of the solvent channel substituent to afford pyridine **14** showed a 10-fold increase in CDK2 activity with a concomitant 10-fold loss of HSF1-stress pathway activity. To probe whether the water-mediated hydrogen bond by the oxygen-linked tail (observed in the co-crystal structure) was essential, propyl-linked **15** was synthesised. However, little change in potency was observed against either CDK2 or the HSF1-stress pathway. Compounds **16–23** were synthesised to investigate the effects on activity in both assays of increased bulk around the amine tail. Both of the *N*-methylpyrrolidine enantiomers (**16** and **17**) and the piperidine **19** displayed modest 2-fold improvement in HSF1-stress pathway activity but maintained similar potency against CDK2, compared with the dimethylamino-solubilising tail **3**.

### 2.3. Combining selected SAR

To assess whether the SAR features described above were additive, the combination compounds **24** and **25** were synthesised (Table 2).

**Table 2:** Analogues which combine best SAR features for HSF1 pathway inhibition

Compound	Structure	HSF1 IC_50_ ^*a*^ (nM)	CDK2 IC_50_ ^*a*^ (nM)
**24**	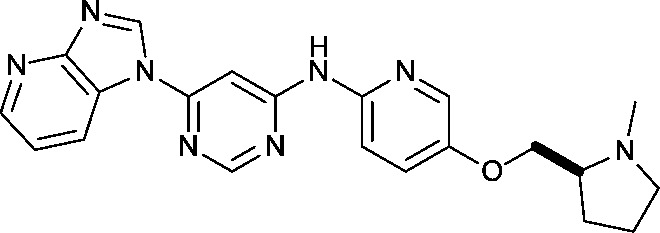	14	146
**25**	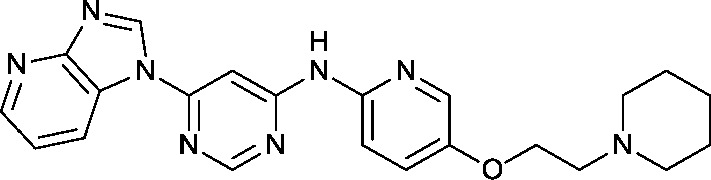	15	55

^*a*^Potency data are reported as the average two determinations.

Modification of the benzimidazole head group to afford a 4-azabenzimidazole in combination with selected cyclised amine tails afforded two potent compounds, methylpyrrolidine **24** and piperidine **25**. Both analogues displayed excellent activity in the HSF1-stress pathway assay (14 nM and 15 nM respectively). Piperidine **25** was also our most active CDK2 inhibitor (55 nM).

## 3. Discussion

**Fig. 3:**
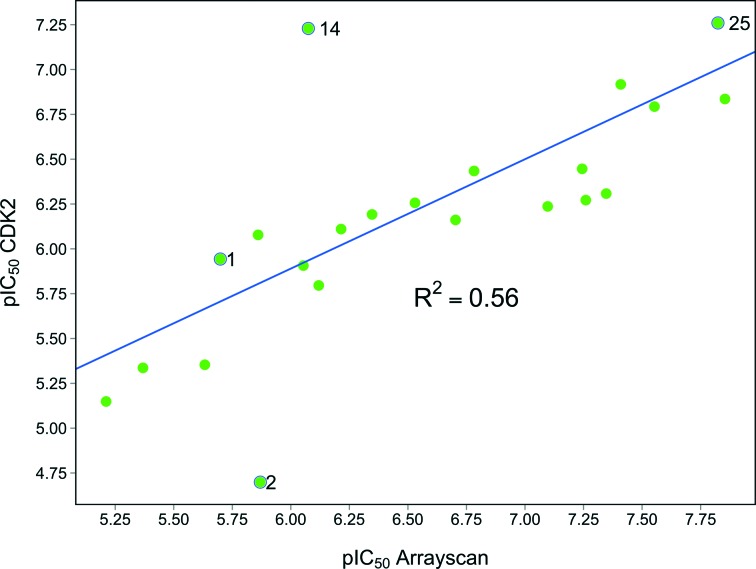
Graph showing the correlation between HSF1 pathway inhibition (ArrayScan™) pIC_50_ and CDK2 inhibition pIC_50_ for compounds in this paper.

With a range of pyrimidines in hand, we were able to analyse the role of CDK2 inhibition in the HSF1 cellular phenotype. Despite a variety of structural changes we observed some correlation (*R* ^2^ = 0.56) between CDK2 activity and HSF1 pathway inhibition (Fig. 3). However, there were analogues which seemed to indicate that CDK2 activity and HSF1 activity could be uncoupled, for example phenyl pyrimidine **2** displays a 15-fold difference in activity between the CDK2 and HSF1-stress pathway assays. This suggests that either inhibition of CDK2 activity is not the key determinant of HSF1-stress pathway antagonism, or alternatively that it is necessary to inhibit multiple kinases (polypharmacology) to observe HSF1-stress pathway inhibition.^25^

It is well established that there is a high level of sequence homology, particularly at the catalytic binding site, between members of the CDK protein family.^26,27^ Consequently, developing selective inhibitors within this protein family is challenging.^26^ If CDK2 is not responsible or is only partially responsible for the observed HSF1-stress pathway inhibition, we hypothesised that the direct target must share considerable structural similarity in the binding site to CDK2.

Therefore 4,6-pyrimidine **3** was submitted to a EMD Millipore CDK panel to assess potency across the selected CDK family members (Table 3). Modest activity was observed against several CDKs; however, this compound was highly potent against CDK9 (14 nM). Following this result, we expanded our screening and several 4,6-disubstituted pyrimidines were also selected for testing against CDK9. In each case the compounds were potent against CDK9 (Table 4), and with the exception of imidazole **6**, activity in the HSF1 assay and CDK9 assay were comparable. Although **6** is the least potent compound in the CDK9 assay the fall off in cellular potency is greater than expected.

**Table 3:** The activity of compound **3** in a CDK family screening panel^*a*^

	CDK1/cyclin B	CDK2/cyclin E	CDK3/cyclin E	CDK5/p35	CDK6/cyclin D3	CDK7/cyclin H/MAT1	CDK9/cyclin T1
IC_50_ (nM)	1800	2800	3500	610	1300	1100	14

^*a*^Data obtained from EMD Millipore screening panel (*N* = 1), since carrying out this screen this CDK assay is now run by Eurofins (http://www.eurofins.com/en.aspx). For assay conditions see ESI.

**Table 4:** Activities of selected 4,6-pyrimidines against CDK9

Compound	HSF1 IC_50_ ^*a*^ (μM)	CDK2 IC_50_ ^*a*^ (μM)	CDK9/cyclin T1^*b*^ (nM)
**3**	0.080	0.580	14
**6**	6.15	7.10	88
**17**	0.045	0.492	8
**19**	0.057	0.358	8
**21**	0.450	0.643	22

^*a*^Potency data are reported as the average of two determinations.

^*b*^Data obtained from EMD Millipore screening panel (*N* = 1), since carrying out this screen this CDK assay is now run by Eurofins (http://www.eurofins.com/en.aspx). For assay conditions see ESI.

CDK9 is known to be a component of the positive transcription elongation factor b (P-TEFb).^28,29^ This enzyme is vital as it stimulates the transcription elongation of most protein coding genes (which include key developmental and stimulus responsive genes) by phosphorylation of the carboxy-terminal domain of RNA polymerase II (RNAPII).^30,31^ Inhibition of CDK9 has been shown to have particular impact on the levels of mRNAs with short half-lives, typified by transcriptionally inducible mRNAs.^32^ It has also been demonstrated that P-TEFb associates with HSF1 and plays a functional role in the stress-induced expression of HSP72.^33^

**Fig. 4:**
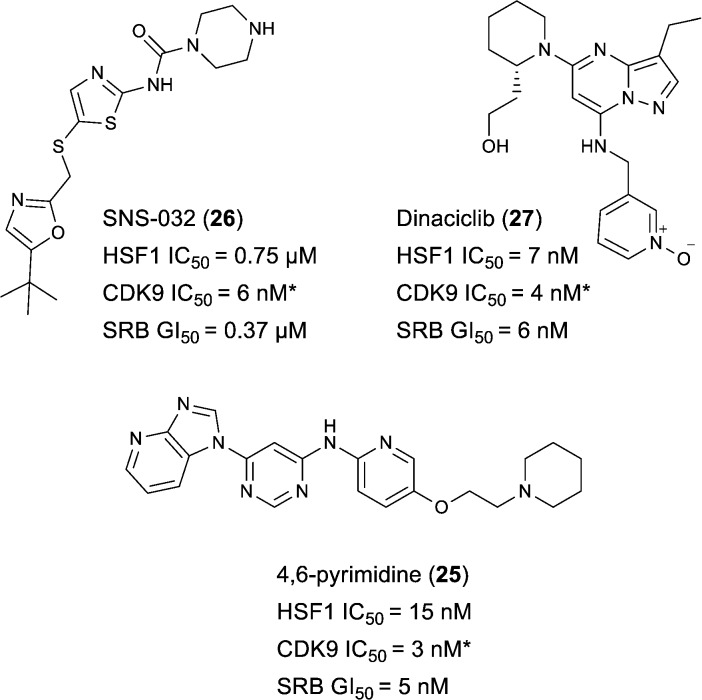
Published CDK9 inhibitors SNS-032 and dinaciclib. Results from cell-ELISA assay for HSF1-mediated HSP72 induction inhibition, CDK9 data from Caliper® biochemical assay and sulforhodamine B (SRB) cell growth inhibition assay. All potency data are reported as the average of two determinations. *At limit of assay sensitivity.

Indeed, Acquaviva *et al.* recently published a screen of drugs and late stage compounds to discover HSF1 transcription inhibitors (defined by the ability of a compound to block induction of HSP72 following treatment with an HSP90 inhibitor, ganetespib).^34^ Amongst the compounds they identified was SNS-032 **26**, a known inhibitor of CDK2, CDK7 and CDK9,^35–37^ which is consistent with the apparent role of CDK9 in the HSF1 inhibition phenotype. The structure of SNS-032 **26** derives from a completely different chemotype to our 4,6-pyrimidine series; therefore it is less likely that both compounds hit a large number of similar protein targets.^38,39^ However, to increase confidence in the role of CDK9 inhibition in our HSF1 pathway inhibition phenotype, we purchased a second structurally distinct CDK9 inhibitor, dinaciclib **27**.^40,41^ In our hands, SNS-032 **26** and dinaciclib **27** displayed CDK9 inhibition values of 6 and 4 nM respectively. Both published CDK9 inhibitors and our optimised 4,6-pyrimidine **25** (CDK9 IC_50_ = 3 nM) were then submitted to a U2OS cell-ELISA assay, which as with the ArrayScan™ assay format, quantifies the ability of a compound to block the induction of HSP72 expression upon cell treatment with 17-AAG. As illustrated in Fig. 4, all three potent CDK9 inhibitors, from distinct chemotypes, are effective inhibitors of the HSF1 pathway. In addition, when these compounds were tested in a sulforhodamine B (SRB) cellular growth inhibition assay as single agents, all three were observed to potently abrogate cell growth.

## 4. Conclusions

A high-throughput phenotypic screen was conducted to find inhibitors of the HSF1-stress pathway in cancer cells and a hit compound based on a di-substituted 4,6-pyrimidine scaffold was discovered. This compound was known to exhibit modest CDK2 activity (1.14 μM). Through the application of SAR and rational design against a CDK2 co-crystal structure, we demonstrated that although there was some correlation between HSF1-stress pathway inhibition and CDK2 inhibition, it was possible to generate analogues where the two measured activities were uncoupled.

Owing to the high homology between the active sites of the CDK family members, 4,6-pyrimidine **3** was screened in a CDK panel and found to be a highly potent inhibitor of CDK9. To test the hypothesis that CDK9 inhibition is involved in HSF1-stress pathway inhibition we purchased two validated CDK9 inhibitors with entirely distinct chemical structures and submitted them to an assay that quantifies HSF1-stress pathway inhibition. Both validated CDK9 inhibitors were potent inhibitors of the HSF1-stress pathway. The rational design of highly selective inhibitors to investigate the function and synergism of different CDKs is extremely challenging. More work is needed to confirm CDK9 as the key molecular target that delivers the HSF1-stress pathway inhibition phenotype and to establish whether kinase polypharmacology is also required. The 4,6-pyrimidine **25** is a useful addition as a structurally distinct *in vitro* chemical tool to study the role of CDK9 inhibition and HSF1-stress pathway inhibition. The broader kinase selectivity profile of 4,6-pyrimidine **25** and the effect on the HSF1 pathway of a number of CDK9 inhibitors, possessing different kinase selectivity profiles, are currently under investigation (a table showing some CDK selectivity data can be found in the ESI‡).

Whilst the disubstituted 4,6-pyrimidine series has shown potency against CDK9 and the HSF1-stress pathway, this series showed high clearance in mouse pharmacokinetic experiments making these compounds unsuitable for progression into animal models. However, other chemical series developed as inhibitors of the HSF1-stress pathway have shown more promise and will be published in due course.

## References

[cit1] Morimoto R. I. (2011). Cold Spring Harbor Symp. Quant. Biol..

[cit2] Anckar J., Sistonen L. (2011). Annu. Rev. Biochem..

[cit3] Vihervaara A., Sistonen L. (2014). J. Cell Sci..

[cit4] Dai C., Whitesell L., Rogers A. B., Lindquist S. (2007). Cell.

[cit5] Workman P., de Billy E. (2007). Nat. Med..

[cit6] Santagata S., Hu R., Lin N. U., Mendillo M. L., Collins L. C., Hankinson S. E., Schnitt S. J., Whitesell L., Tamimi R. M., Lindquist S., Ince T. A. (2011). Proc. Natl. Acad. Sci. U. S. A..

[cit7] Dai C., Dai S., Cao J. (2012). J. Cell. Physiol..

[cit8] Dai C., Sampson S. B. (2016). Trends Cell Biol..

[cit9] Mendillo M. L., Santagata S., Koeva M., Bell G. W., Hu R., Tamimi R. M., Fraenkel E., Ince T. A., Whitesell L., Lindquist S. (2012). Cell.

[cit10] Natalia V., Agnieszka T., Wieslawa W. (2014). Curr. Cancer Drug Targets.

[cit11] Xi C., Hu Y., Buckhaults P., Moskophidis D., Mivechi N. F. (2012). J. Biol. Chem..

[cit12] Fang F., Chang R., Yang L. (2012). Cancer.

[cit13] Scott K. L., Nogueira C., Heffernan T. P., van Doorn R., Dhakal S., Hanna J. A., Min C., Jaskelioff M., Xiao Y., Wu C.-J., Cameron L. A., Perry S. R., Zeid R., Feinberg T., Kim M., Vande Woude G., Granter S. R., Bosenberg M., Chu G. C., DePinho R. A., Rimm D. L., Chin L. (2011). Cancer Cell.

[cit14] Scherz-Shouval R., Santagata S., Mendillo M. L., Sholl L. M., Ben-Aharon I., Beck A. H., Dias-Santagata D., Koeva M., Stemmer S. M., Whitesell L., Lindquist S. (2014). Cell.

[cit15] Brandvold K. R., Morimoto R. I. (2015). J. Mol. Biol..

[cit16] Smith H. L., Li W., Cheetham M. E. (2015). Semin. Cell Dev. Biol..

[cit17] Doyle S. M., Genest O., Wickner S. (2013). Nat. Rev. Mol. Cell Biol..

[cit18] Tang Z., Dai S., He Y., Doty R. A., Shultz L. D., Sampson S. B., Dai C. (2015). Cell.

[cit19] Chen Y., Chen J., Loo A., Jaeger S., Bagdasarian L., Yu J., Chung F., Korn J., Ruddy D., Guo R., McLaughlin M. E., Feng F., Zhu P., Stegmeier F., Pagliarini R., Porter D., Zhou W. (2013). Oncotarget.

[cit20] de Billy E., Powers M. V., Smith J. R., Workman P. (2009). Cell Cycle.

[cit21] West J. D. Wang Y. Morano K. A., Chem. Res. Toxicol., 2012, 25 , 2036 –2053 , . We have also used AUY922, a non-quinone HSP90 inhibitor to stimulate an HSF1-mediated response in our assay with comparable results . 22799889 10.1021/tx300264x PMC3472121

[cit22] Banerji U., Walton M., Raynaud F., Grimshaw R., Kelland L., Valenti M., Judson I., Workman P. (2005). Clin. Cancer Res..

[cit23] Chohan T. A., Qian H., Pan Y., Chen J. Z. (2015). Curr. Med. Chem..

[cit24] Cho Y. S., Borland M., Brain C., Chen C. H. T., Cheng H., Chopra R., Chung K., Groarke J., He G., Hou Y., Kim S., Kovats S., Lu Y. P., O'Reilly M., Shen J. Q., Smith T., Trakshel G., Vogtle M., Xu M., Sung M. J. (2010). J. Med. Chem..

[cit25] Knight Z. A., Lin H., Shokat K. M. (2010). Nat. Rev. Cancer.

[cit26] Asghar U., Witkiewicz A. K., Turner N. C., Knudsen E. S. (2015). Nat. Rev. Drug Discovery.

[cit27] Echalier A., Hole A. J., Lolli G., Endicott J. A., Noble M. E. (2014). ACS Chem. Biol..

[cit28] Canduri F., Perez P. C., Caceres R. A., de Azevedo, Jr. W. F. (2008). Med. Chem..

[cit29] Krystof V., Baumli S., Furst R. (2012). Curr. Pharm. Des..

[cit30] Marshall N. F., Peng J., Xie Z., Price D. H. (1996). J. Biol. Chem..

[cit31] Guo J., Price D. H. (2013). Chem. Rev..

[cit32] Lapenna S., Giordano A. (2009). Nat. Rev. Drug Discovery.

[cit33] Zhang Y., Murshid A., Prince T., Calderwood S. K. (2011). PLoS One.

[cit34] Acquaviva J., He S., Sang J., Smith D. L., Sequeira M., Zhang C., Bates R. C., Proia D. A. (2014). Mol. Cancer Res..

[cit35] Chen R., Wierda W. G., Chubb S., Hawtin R. E., Fox J. A., Keating M. J., Gandhi V., Plunkett W. (2009). Blood.

[cit36] Conroy A., Stockett D., Walker D., Arkin M., Hoch U., Fox J., Hawtin R. (2009). Cancer Chemother. Pharmacol..

[cit37] Tong W.-G., Chen R., Plunkett W., Siegel D., Sinha R., Harvey R. D., Badros A. Z., Popplewell L., Coutre S., Fox J. A., Mahadocon K., Chen T., Kegley P., Hoch U., Wierda W. G. (2010). J. Clin. Oncol..

[cit38] Haupt V. J., Daminelli S., Schroeder M. (2013). PLoS One.

[cit39] Milletti F., Vulpetti A. (2010). J. Chem. Inf. Model..

[cit40] Parry D., Guzi T., Shanahan F., Davis N., Prabhavalkar D., Wiswell D., Seghezzi W., Paruch K., Dwyer M. P., Doll R., Nomeir A., Windsor W., Fischmann T., Wang Y., Oft M., Chen T., Kirschmeier P., Lees E. M. (2010). Mol. Cancer Ther..

[cit41] Paruch K., Dwyer M. P., Alvarez C., Brown C., Chan T.-Y., Doll R. J., Keertikar K., Knutson C., McKittrick B., Rivera J., Rossman R., Tucker G., Fischmann T., Hruza A., Madison V., Nomeir A. A., Wang Y., Kirschmeier P., Lees E., Parry D., Sgambellone N., Seghezzi W., Schultz L., Shanahan F., Wiswell D., Xu X., Zhou Q., James R. A., Paradkar V. M., Park H., Rokosz L. R., Stauffer T. M., Guzi T. J. (2010). ACS Med. Chem. Lett..

