# Supplementary material for: Discovery of 4,6-disubstituted pyrimidines as potent inhibitors of the heat shock factor 1 (HSF1) stress pathway and CDK9
Source: Medchemcomm. 2016 Jun 13;7(8):1580–6. doi: 10.1039/c6md00159a (PMC5048338; doi:10.1039/c6md00159a)
Supplement: Supplementary file 1 [file MD-007-C6MD00159A-s001.pdf]

Electronic Supplementary Material (ESI) for MedChemComm.
This journal is © The Royal Society of Chemistry 2016

Supporting Information

Discovery of 4,6-disubstituted pyrimidines as potent inhibitors of the Heat Shock Factor (HSF1) stress pathway and CDK9.

Carl S. Rye,[a§] Nicola E. A. Chessum,[a§] Scott Lamont,[b] Kurt G. Pike,[b] Paul Faulder,[b] Julie Demeritt,[b] Paul Kemmitt,[b] Julie Tucker,[b,d] Lorenzo Zani,[a,c] Matthew D. Cheeseman,[a] Rosie Isaac,[b] Louise Goodwin,[b] Joanna Boros,[b] Florence Raynaud,[a] Angela Hayes,[a] Alan T. Henley,[a] Emmanuel de Billy,[a] Christopher J. Lynch,[a] Swee Y. Sharp,[a] Robert te Poele,[a] Lisa O' Fee,[a] Kevin M. Foote,[b] Stephen Green,[b] Paul Workman,[a] and Keith Jones[a]**

[a]Cancer Research UK Cancer Therapeutics Unit at The Institute of Cancer Research, London SW7 3RP, United Kingdom. [b]AstraZeneca, Alderley Park, Macclesfield, Cheshire, SK10 4TG, United Kingdom. [c]Current address: Institute for the Chemistry of Organometallic Compounds (CNR-ICCOM), Via Madonna del Piano 10, 50019 Sesto Fiorentino, Italy. [d]Current address: Northern Institute for Cancer Research, Paul O'Gorman Building, Medical School, Newcastle University, Framlington Place, Newcastle upon Tyne, NE2 4HH, United Kingdom.

Table of Contents:

ArrayScan HSP72 protein assay…………………………………………………. S2

CDK2 enzyme assay………………………………………………………….. S2

Cell-Based ELISA assay………………………………………………........ S3

Eurofins CDK panel assays…………………………………………………… S3

Caliper CDK9 assay……………………………………………………………S4

SRB cell growth inhibition assay………………………………………………S4

Table showing ratios observed between CDK2/7/9 for key compounds………S5

CDK2-**4** crystal structure determination………………………………………S6

Synthetic Schemes…………………………………………………………….. S8

General Experimental………………………………………………………. S12

Experimental procedures and compound characterization……………………. S13

ArrayScan HSP72 protein assay

U2OS cells (1500 per well) were plated in 40 µL of DMEM media (containing 10% fetal calf serum and 2 mM Glutamax T-1) in costar 384-well plates and left overnight at 37 °C and 5% CO_2 to adhere. Cells were then dosed with compound diluted in DMSO (120 nL added to each well to give 0.0313−30 µM concentrations of compound) and incubated at 37 °C and 5% CO_2. After 1 h of treatment with compound, all wells except min wells were dosed with 17AAG diluted in DMSO (10 nL added to each well to give 250 nM final concentration) and plates were incubated overnight at 37 °C and 5% CO_2. The following day the cells were fixed by addition of 20 µL/well of 12% formaldehyde with 1:1700 Hoechst in PBS for 10 min at room temperature. The fixative was decanted and the wells washed once with 50 µL of phosphate buffer saline (PBS).The PBS was subsequently aspirated, and the cells were permeabilized by addition of 20 µL/well of PBS 0.3% Triton X-100 for 20 min at room temperature. The wells were then washed with 80 µL of PBS prior to the addition of 20 µL of combined primary and secondary antibodies diluted in PBS (1:10 000 mouse anti-Hsp72 #SPA-810 purchased from Stressgen and 1:3000 Alexa Fluor 488 goat anti-mouse IgG (H+L) #A-11001 molecular probes), for 2 h at room temperature. The wells were then washed with 50 µL of PBS. Finally, 50 µL of PBS was added to each well, and the plates were sealed ready to analyze. Analysis was carried out using a Cellomics Arrayscan VTI instrument and the Cellomics Arrayscan Compartmental Analysis algorithm to measure cellular levels of HSP72.

CDK2 enzyme assay: Off-chip Mobility SHIFT Assay

All reagents were purchased from Sigma-Aldrich unless otherwise specified. Inhibition of CDK2/cyclinE activity was quantified using an off-chip incubation, mobility shift assay, which uses a microfluidic chip (Caliper Life Sciences) to measure the conversion of a fluorescent peptide substrate to phosphorylated product. An Off-chip Mobility Shift Chip with 12 sippers (Caliper Life Sciences) was prepared for use according to the manufacturer’s instructions using Coating Reagent #3 (Caliper Life Sciences). The off-chip incubation was carried out in white, 384-well, flat bottom, low volume assay plates (Greiner). 5 µL peptide mix (1.5 µM substrate peptide (fluorescein-QSPKKG-CONH2, Bachem) and 90 µM ATP in kinase base buffer (50 mM HEPES, pH7.5 (Calbiochem), 0.06% CHAPSO)) were added to each well of the prepared compound plates which contained 120 nL compound stock solution per well. The reaction was then initiated by the addition of 6 µL enzyme mix (1.0 nM CDK2/cyclin E (Upstate/Millipore), 1.0 mM DTT and 5.0 mM $MgCl_2$ in kinase base buffer). After incubation for 60 min at room temperature, each reaction was terminated by the addition of 10µL of stop solution containing 100 mM HEPES pH 7.5 (Calbiochem), 0.033% Brij-35, 0.22% Coating Reagent #3 (Caliper Life Sciences), 44 mM EDTA and 5 % DMSO. Reaction products were analysed using a Caliper LabChip LC3000 instrument and peak height was used to calculate percentage conversion of substrate to product.

For compound response testing, a 12 point compound concentration range was used. Compounds were acoustically dosed (Echo 555; Labcyte) into assay-ready plates using 11 half log intervals from 100 µM to 0.1 µM plus a 12th point at 0.01 µM. Each well was backfilled with the required volume up to 120 nL of 100% DMSO to ensure a final 1% v/v DMSO concentration in the assay. In addition to the compound test wells, each plate carried maximum and minimum controls. Staurosporine at 10 µM was used to inhibit CDK2 activity for the minimum control. As a maximum control, 120 nL pure DMSO was added to the wells. Each plate contained at least 11 randomly distributed maximum and minimum controls. After the addition of compounds to the assay plates, the assay was performed as detailed above. Percentage conversion of substrate to product was used to calculate the effect of each compound on the kinase activity of CDK2/cyclinE. Enzyme inhibition model 3, non-linear curve fit analysis within OriginLab™ software was used to fit dose response curves and estimate the

concentration of compound required to reduce the enzyme activity to 50% of the conversion calculated from an average of the maximum controls.

Cell-based ELISA (Cellisa) for HSP72 expression

To follow HSP72 protein expression, a product of HSF1 transcriptional activity, Cellisa was developed. Cells (5-8x10^4cells/ml) were seeded into 96-well plates and incubated at 37 ^{o}C for 48 h. Compounds were then added at a range of concentrations and incubated for 1 h before addition of 17-AAG (250 nM). Cells were then incubated for 18 h. The medium was removed and cells were fixed with fixing solution (4% paraformaldehyde, 0.3% TritonX-100 in PBS) for 30 min at 4 ^{o}C. The plates were then washed with 0.1% Tween-20/deionised water before blocking with 5% milk for 30 min at 37 ^{o}C. After washing the plates, HSP72 antibody (SPA-810, Enzo Life) was added for 1.5 h at 37 ^{o}C. Following 4 x washes, the plates were incubated with europium-labelled anti-mouse antibody (0.6ug/ml) in Delfia assay buffer (Perkin Elmer) for 1 h at 37 ^{o}C. After washing the plates, Delfia enhancement solution was added, shaken for 10 min before reading in the Envision plate reader (Perkin-Elmer) with excitation at 340 nm and emission at 615 nm. The plates were washed again before protein determination using the BCA assay. The europium counts were normalised for the amount of protein in each well. The 50% inhibitory concentration value of the compound was then calculated and presented.

Eurofins CDK panel assay conditions

For the screening conditions and assay protocol for each of these assays see https://www.eurofinspanlabs.com/Catalog/AssayCatalog/AssayCatalog.aspx?path=164&leaf=164&track=Add%2f2%2fTarget+Class%2fKinase&_ga=1.62213996.1760850026.1408527181

CDK1/cyclinB (h)

CDK1/cyclinB (h) is incubated with 8 mM MOPS pH 7.0, 0.2 mM EDTA, 0.1 mg/mL histone H1, 10 mM MgAcetate and [9-33P]-ATP (specific activity approx. 500 cpm/pmol, concentration as required). The reaction is initiated by the addition of the MgATP mix. After incubation for 40 minutes at room temperature, the reaction is stopped by the addition of 3% phosphoric acid solution. 10 μL of the reaction is then spotted onto a P30 filtermat and washed three times for 5 minutes in 75 mM phosphoric acid and once in methanol prior to drying and scintillation counting.

CDK2/cyclinE (h)

CDK2/cyclinE (h) is incubated with 8 mM MOPS pH 7.0, 0.2 mM EDTA, 0.1 mg/mL histone H1, 10 mM MgAcetate and [9-33P]-ATP (specific activity approx. 500 cpm/pmol, concentration as required). The reaction is initiated by the addition of the MgATP mix. After incubation for 40 minutes at room temperature, the reaction is stopped by the addition of 3% phosphoric acid solution. 10 μL of the reaction is then spotted onto a P30 filtermat and washed three times for 5 minutes in 75 mM phosphoric acid and once in methanol prior to drying and scintillation counting.

CDK3/cyclinE (h)

CDK3/cyclinE (h) is incubated with 8 mM MOPS pH 7.0, 0.2 mM EDTA, 0.1 mg/mL histone H1, 10 mM Mg Acetate and [9-33P]-ATP (specific activity approx. 500 cpm/pmol, concentration as required). The reaction is initiated by the addition of the MgATP mix. After incubation for 40 minutes at room temperature, the reaction is stopped by the addition of 3% phosphoric acid solution. 10 μL of the reaction is then spotted onto a P30 filtermat and washed three times for 5 minutes in 75 mM phosphoric acid and once in methanol prior to drying and scintillation counting.

CDK5/p35 (h)

CDK5/p35 (h) is incubated with 8 mM MOPS pH 7.0, 0.2 mM EDTA, 0.1 mg/mL histone H1, 10 mM Mg Acetate and [9-33P]-ATP (specific activity approx. 500 cpm/pmol, concentration as required). The reaction is initiated by the addition of the MgATP mix. After incubation for 40 minutes at room temperature, the reaction is stopped by the addition of 3% phosphoric acid solution. 10 μL of the reaction is then spotted onto a P30 filtermat and washed three times for 5 minutes in 75 mM phosphoric acid and once in methanol prior to drying and scintillation counting.

CDK6/cyclinD3 (h)

CDK6/cyclinD3 (h) is incubated with 8 mM MOPS pH 7.0, 0.2 mM EDTA, 0.1 mg/mL histone H1, 10 mM MgAcetate and [9-33P]-ATP (specific activity approx. 500 cpm/pmol, concentration as required). The reaction is initiated by the addition of the MgATP mix. After incubation for 40 minutes at room temperature, the reaction is stopped by the addition of 3% phosphoric acid solution. 10 μL of the reaction is then spotted onto a P30 filtermat and washed three times for 5 minutes in 75 mM phosphoric acid and once in methanol prior to drying and scintillation counting.

CDK7/cyclinH/MAT1 (h)

CDK7/cyclinH/MAT1 (h) is incubated with 8 mM MOPS pH 7.0, 0.2 mM EDTA, 500 μM peptide, 10 mM MgAcetate and [9-33P]-ATP (specific activity approx. 500 cpm/pmol, concentration as required). The reaction is initiated by the addition of the MgATP mix. After incubation for 40 minutes at room temperature, the reaction is stopped by the addition of 3% phosphoric acid solution. 10 μL of the reaction is then spotted onto a P30 filtermat and washed three times for 5 minutes in 75 mM phosphoric acid and once in methanol prior to drying and scintillation counting.

CDK9/cyclinT1 (h)

CDK9/cyclinT1 (h) is incubated with 8 mM MOPS pH 7.0, 0.2 mM EDTA, 100 μM KTFCGTPEYLAPEVRREPRILSEEEQEMFRDFDYIADWC, 10 mM MgAcetate and [9-33P]-ATP (specific activity approx. 500 cpm/pmol, concentration as required). The reaction is initiated by the addition of the MgATP mix. After incubation for 40 minutes at room temperature, the reaction is stopped by the addition of 3% phosphoric acid solution. 10 μL of the reaction is then spotted onto a P30 filtermat and washed three times for 5 minutes in 75 mM phosphoric acid and once in methanol prior to drying and scintillation counting.

Caliper Assay for CDK9 activity

CDK9 Kinase activity and determination of IC_{50} values was carried out using a microfluidic format that monitors the separation of a phosphorylated product from its substrate. The assay was run on an EZ Reader II (PerkinElmer, MA, USA) using 1.5 µM fluorescently labelled peptide (Perkin Elmer, Peptide 34, Cat No.760643, Sequence 5'FAM-RRRFRPASPLRGPPK-COOH), 0.5ng/µL CDK9/CycT1(Carna Biosciences Prod. No.04-110), 15 µM ATP (the determined Km^{app} for ATP) and 1% DMSO, incubated at room temperature for 30 min. IC_{50} values were calculated using a 4 parameter logistics fit using the Studies package from Dotmatics.

SRB cell growth inhibition assay

Cell growth inhibition was measured by the sulforhodamine B (SRB) assay.[1] Cells (U2OS, 5 x 10^3 cells/mL) were seeded into 96-well microtitre plates and left at 37 °C overnight to allow the cells to attach. Compounds at a range of concentrations were added to quadruplicate wells for 96 h. The cells were then fixed with 10% tricholoroacetic acid and stained with 0.4% SRB in 1% acetic acid. The

GI_{50} values were determined as the compound concentration that reduced absorption to 50% of that in the untreated control wells.

Table showing ratios of activity observed between CDK2/7/9 for key compounds

Compound	CDK2/CyclinE	CDK7/CyclinH/MAT1	CDK9/CyclinT1
**3**	200[a]	79[a]	1[a]
**25**	20[b]	38[c]	1[c,d]
**SNS-032**[e]	10	16	1
**Dinaciclib**[f]	1	*	4
**LY2857785**[g]	*	22	1

Results in nM. [a] Result obtained from EMD Millipore Screening Panel. [b]Result obtained from Z-Lyte assay, Life Technologies. [c]Result obtained from Adapta screen assay, Life Technologies. [d]Below limit of assay detection. Details for Life Technologies assays may be found at: http://www.thermofisher.com/uk/en/home/industrial/pharma-biopharma/drug-discovery-development/target-and-lead-identification-and-validation/kinasebiology/kinase-activity-assays.html

[e]Data from reference 35.

[f]Data from reference 40.

[g]Data from T.Yin, M.J. Lallena, E.M. Kreklau, K.R. Fales, S. Carballares, R. Torres, G.N. Wishart, R.T. Ajamie, D. M. Cronier, P.W. Iversen, T.I. Meier, R.T. Foreman, D. Zeckner, S.E. Sissons, B.W. Halstead, A.B. Lin, G.P. Donoho, Y. Quian, S. Li, S. Wu, A. Aggarwal, X.S. Ye, J.J. Starling, R.B. Gaynor, A. De Dios, J. Du. *Mol. Cancer Ther.*, 2014, 13, p.1442-1456.

* Data not available.

*CDK2-**4** crystal structure determination*

Protein and crystals were obtained according to established procedures.[2, 3] The CDK2 used for crystallization was expressed, purified and crystallised in the absence of cyclin. Crystals were cross-linked[4] by incubation in mother liquor containing 0.14% glutaraldehyde for 15 min before soaking in 20 mM compound **4** for 70 h in mother liquor containing 20% DMSO. Crystals were flash cooled in a stream of nitrogen gas at 100K (Oxford Cryostream) prior to diffraction data collection at 100K using CuKα radiation from a Rigaku FRE rotating anode generator equipped with VariMaxHF optics and a Saturn944 CCD detector and a Rigaku XStream cryo-cooling system. Data were integrated and scaled using XDS[5] and SCALA[6] as implemented within autoPROC.[7] Data reduction and structure solution by molecular replacement were carried out using programs from the CCP4[8] suite. Compound X was modeled into the electron density using Flynn.[9] The protein-compound complex model was refined using Refmac5 v5.0109,[10] interspersed with rounds of manual model building in Coot.[11] The final structure[12] has been deposited in the Protein Data Bank with the deposition code 4bzd together with structure factors and detailed experimental conditions. Detailed statistics of the data collection and final model are presented in Table S1.

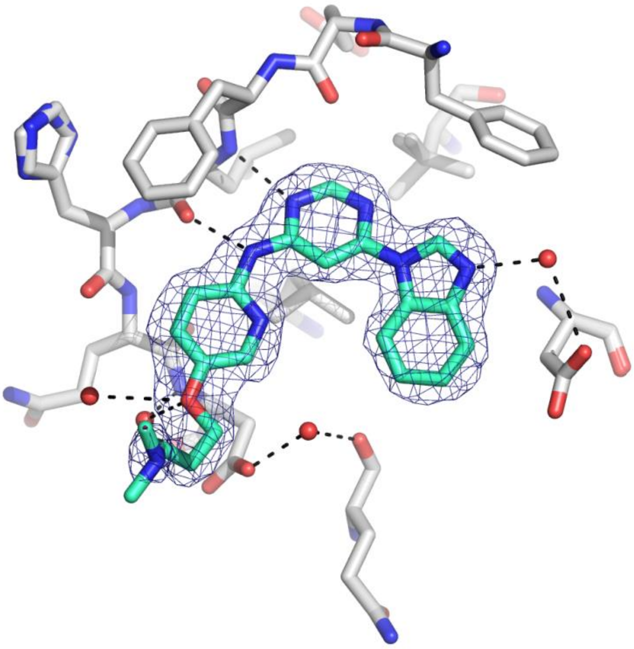

Figure S1. Crystal structure of CDK2 in complex with compound **4** showing final $2F_o$ - F_c electron density for the compound **4** (blue, 1.0σ level). Selected nearby protein residues are shown. Hydrogen bonding interactions with the protein and selected water molecules are indicated as dashed black lines. The figure was prepared using PyMol.[13]

Table S1. X-ray Diffraction Data for CDK2-**4** crystal structure

**X-ray Diffraction Data-Processing and Refinement Statistics**	
Space group	$P2_12_12_1$
Cell constants a; b; c (Å)	53.4; 71.9; 72.3; 90.0; 90.0; 90.0
Resolution range (Å)[a]	50.97-1.83 (1.88-1.83)
Completeness overall (%)[a]	92.2 (61.9)
Reflections, unique	23070
Multiplicity [a]	4.44 (1.8)
Mean(I)/sd(I) [a]	17.1 (1.5)
Rmerge $_{overall}$[a,b]	0.048 (0.467)
Rvalue $_{overall}$ (%)[a,c]	17.33 (29.4)
Rvalue $_{free}$ (%)[a]	20.40 (29.6)
Non hydrogen protein atoms	2224
Non hydrogen ligand atoms	29
Solvent molecules	216
R.m.s. deviations from ideal values	
Bond lengths (Å)	0.015
Bond angles (°)	1.436
Average B values (Å^2)	
Protein main chain atoms	25.742
Protein all atoms	26.366
Ligand	39.269
Solvent	35.634
Φ, Ψ angle distribution for residues[d]	
In most favoured regions (%)	91.6
In additional allowed regions (%)	7.6
In generously regions (%)	0.4
In disallowed regions (%)	0.4

[a]Values in parentheses refer to the outer resolution shell

[b]$R_{merge} = S_{hkl} [(\Sigma_i |I_i - \langle I \rangle|)/ \Sigma_i I_i]$

[c]$R_{value} = S_{hkl} ||F_{obs}| - |F_{calc}|| / S_{hkl} |F_{obs}|$

R_{free} is the cross-validation R factor computed for the test set of 5% of unique reflections

[d]Ramachandran statistics as defined by PROCHECK[14]

Synthetic Schemes

Scheme 1. Synthesis of Compound 2[a]

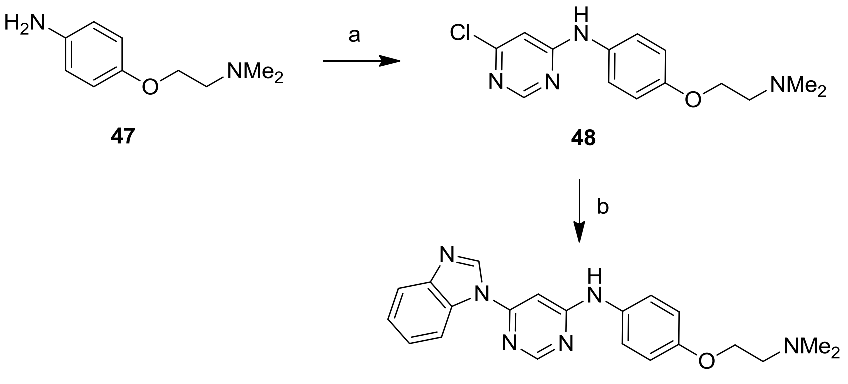

2

[a]Reagents and conditions: (a) 4,6-dichloropyrimidine, AcOH/H_2O (5:1), rt, 17%; (b) benzimidazole, NaH, DMF, 100 °C, 16%.

The synthesis of compound **2** is depicted in Scheme 1. Starting with aniline **47**,[15] an acid-catalysed S_NAr reaction with 4,6-dichloropyrimidine afforded the chloropyrimidine **48**. An S_NAr reaction using sodium hydride and benzimidazole to displace the remaining chlorine afforded the desired 4,6-pyrimidine phenyl analogue **2**.

Scheme 2. Synthesis of Compounds 6-13.[a] Variation of the benzimidazole.

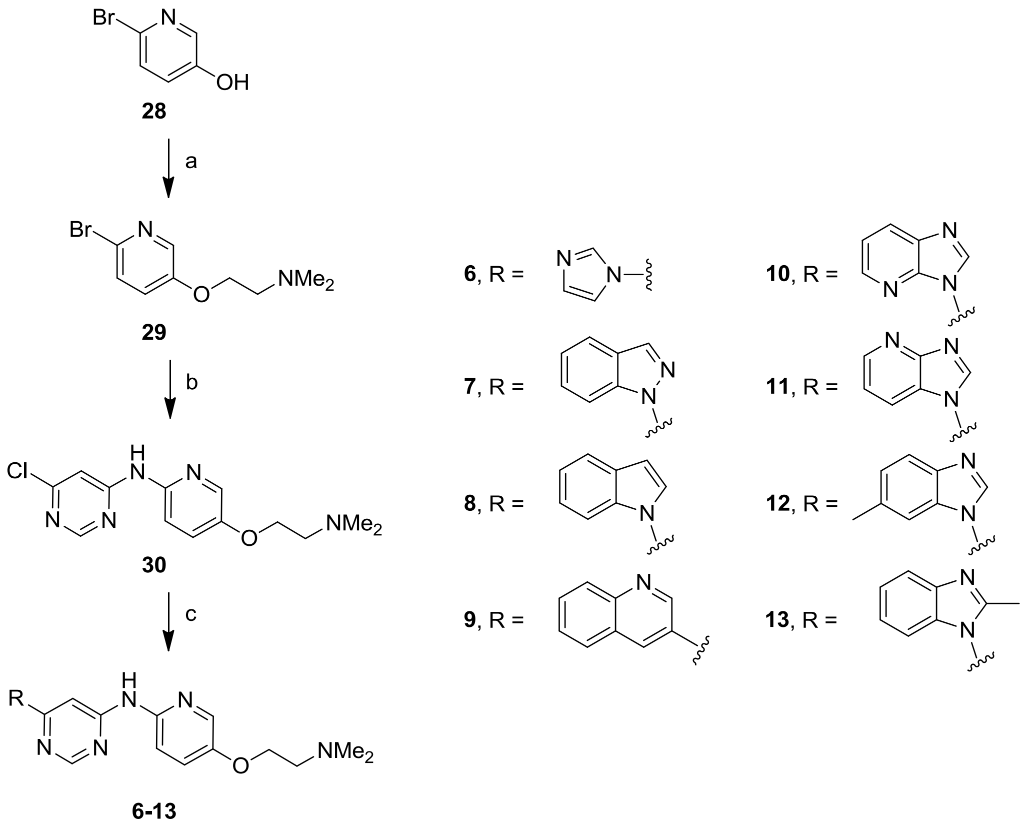

[a]Reagents and conditions: (a) 2-dimethylaminoethyl chloride hydrochloride, Cs_2CO_3, acetone, reflux, 95%; (b) 4-amino-6-chloropyrimidine, $Pd(OAc)_2$, Xantphos, Cs_2CO_3, dioxane, 105 °C, 43%; (c) RH, NaH, DMF, 100 °C (or 120 °C), 8-60% (**6, 8, 10-12**), or RH, Pd_2dba_3, Xantphos, NaO^tBu, toluene, 100 °C, 5-21% (**7, 13**), or $RB(OH)_2$, $Pd(PPh_3)_2Cl_2$, K_2CO_3, TBAB, dioxane, 90 °C, 72% (**9**).

The synthesis of compounds **6-13** is outlined in Scheme 2. Alkylation of 2-bromo-5-hydroxypyridine (**28**) with 2-dimethylaminoethyl chloride hydrochloride, followed by a palladium-mediated coupling with 4-amino-6-chloropyrimidine produced the common intermediate **29** in a moderate yield. The majority of the *N*-linked compounds were synthesized by an S_NAr reaction facilitated by deprotonating the appropriate heterocycle with sodium hydride followed by heating with compound **30** in DMF at 100 °C. The azabenzimidazole regioisomers **10** and **11** were easily separated by silica gel chromatography with the 4-azabenzimidazole being the major isomer formed. On the other hand, the separation of the methylbenzimidazole **12** and the corresponding undesired regioisomer was problematic on silica gel, and was accomplished by HPLC purification. The indazole compound **7** was isolated in a low yield *via* a Buchwald-Hartwig type coupling after separation of the regioisomeric *N*2-linked product using HPLC purification. The methylbenzimidazole **13** was also synthesized using Buchwald-Hartwig conditions. The quinoline **9** was synthesised in reasonable yield *via* a Suzuki cross-coupling from the appropriate boronic acid.

Scheme 3. Synthesis of Compounds 3, 4, 16, 18-23.[a] Variation of the basic amine tail.

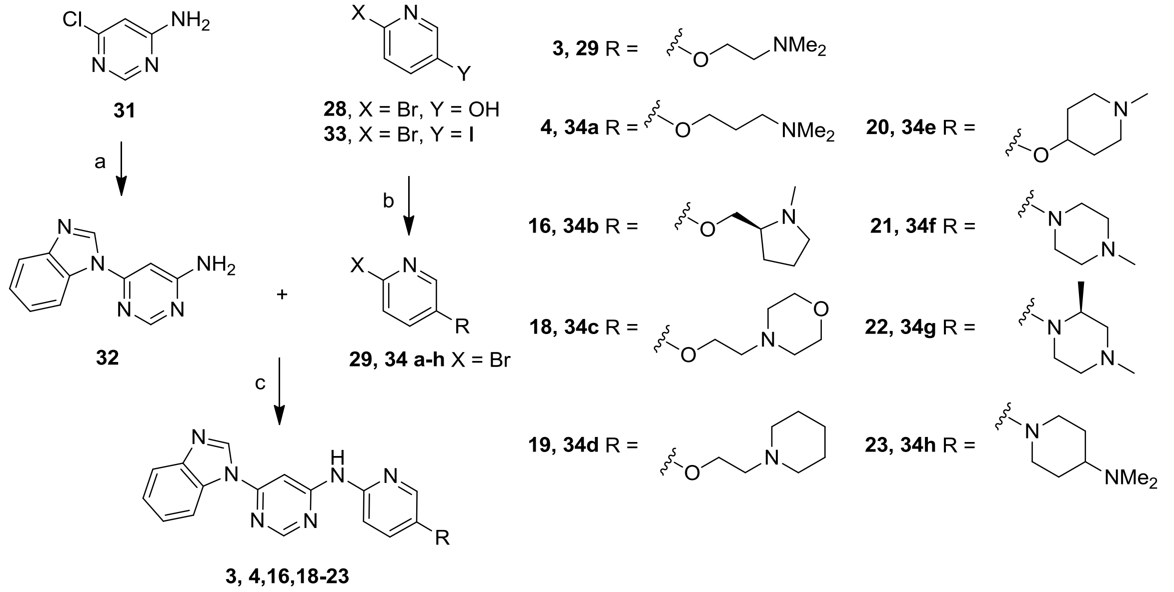

[a]Reagents and conditions: (a) benzimidazole, NaH, DMF, 100 °C, 78%; (b) for **29**: from **28**, 2-dimethylaminoethyl chloride hydrochloride, Cs_2CO_3, acetone, reflux, 95%, for **34a,c,d**: from **28**, appropriate alkyl chloride, K_2CO_3, DMF, 70 °C, 55-86%, for **34b,e**: from **28**, appropriate alcohol, diisopropyl azodicarboxylate, PPh_3, THF, 0 °C to rt, 53-59%, for **34f,g,h** (from **33**) and RH, Pd_2dba_3, Xantphos, NaO^{t}Bu, toluene, 100 °C, 24-70%; (c) for **3, 4, 16, 18-20**: Pd_2dba_3, Xantphos, Cs_2CO_3, dioxane, 100 °C, 13-58%, for **21-23**: Pd_2dba_3, Xantphos, NaO^{t}Bu, toluene, 100 °C, 56-64%.

The syntheses of the compounds where the *O*- and *N*-linked basic amine tails are varied are shown in Scheme 3. The majority of the *O*-linked compounds, as outlined in Scheme 3, were generated by the *O*-alkylation of 2-bromo-5-hydroxy pyridine (**28**) with either the appropriate alkyl halide (for compounds **34a-e**) or *via* a Mitsunobu reaction with the appropriate alcohol (for compounds **34b,e**). The *N*-linked tails were installed *via* palladium-mediated cross-coupling reactions with the appropriate amine and 2-bromo-5-iodopyridine (**33**) (for compounds **34f-h**). The desired final products were then synthesised *via* another palladium-mediated cross-coupling between the aminopyrimidine **32** (which was constructed in good yield using a sodium hydride-mediated S_NAr reaction between 2-chloro-4-aminopyrimidine and benzimidazole) and the corresponding side chain **29**, **34a-h**.

Scheme 4. Synthesis of Compounds 14 and 15.[a]

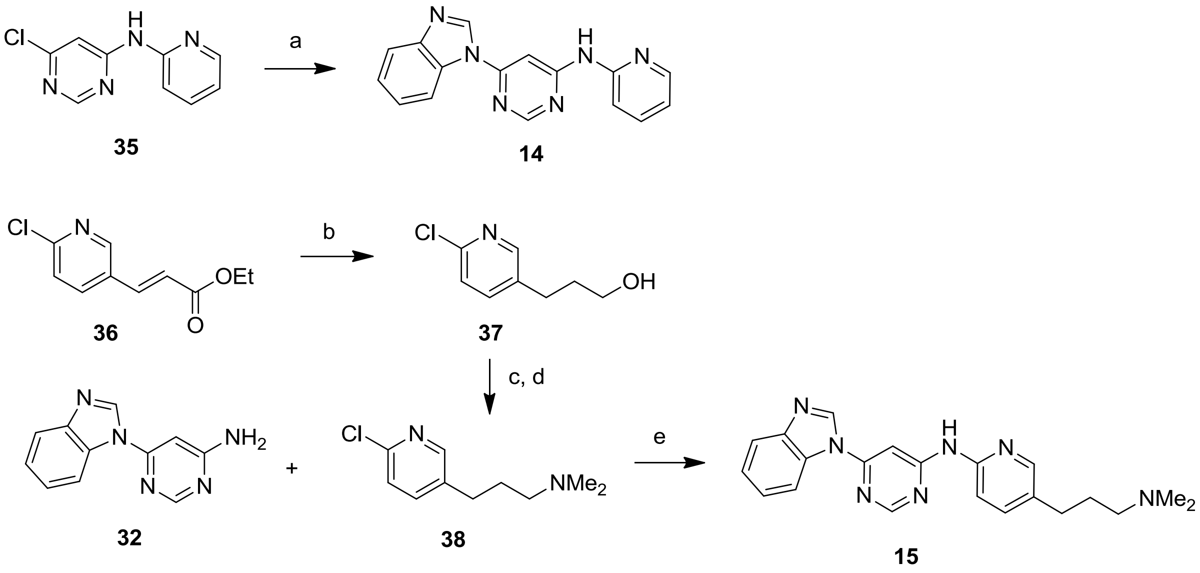

[a]Reagents and conditions: (a) benzimidazole, NaH, DMF, 110 °C, 83%; (b) $LiAlH_4$, Et_2O, 0 °C to rt, 15%; (c) methanesulfonyl chloride, diisopropylethylamine, CH_2Cl_2, 0 °C; then, (d) dimethylamine, THF, reflux, 46%; (e) Pd_2dba_3, Xantphos, NaO^tBu, toluene, 100 °C, 71%.

Scheme 4 outlines the synthesis of the compounds where there is either no amine tail, or the tail is linked *via* carbon to the pyridine ring. The synthesis of the derivative without any tail on the pyridine ring (**14**) was accomplished in good yield *via* a S_NAr reaction between benzimidazole and the chloropyrimidine **35**.[16] The carbon-linked tail analogue **15** of the parent compound was synthesized beginning with the reduction of 3-(6-chloropyridin-3-yl)acrylic acid ethyl ester (**36**) using lithium aluminium hydride to produce alcohol **37**,[17] although in a yield of only of 15%. After conversion of this alcohol to the mesylate, the reaction with dimethylamine furnished compound **38**. Palladium mediated cross-coupling of **38** with amino pyrimidine **32** afforded compound **15** in good yield.

Scheme 5. Synthesis of Compound 5 and 17.[a]

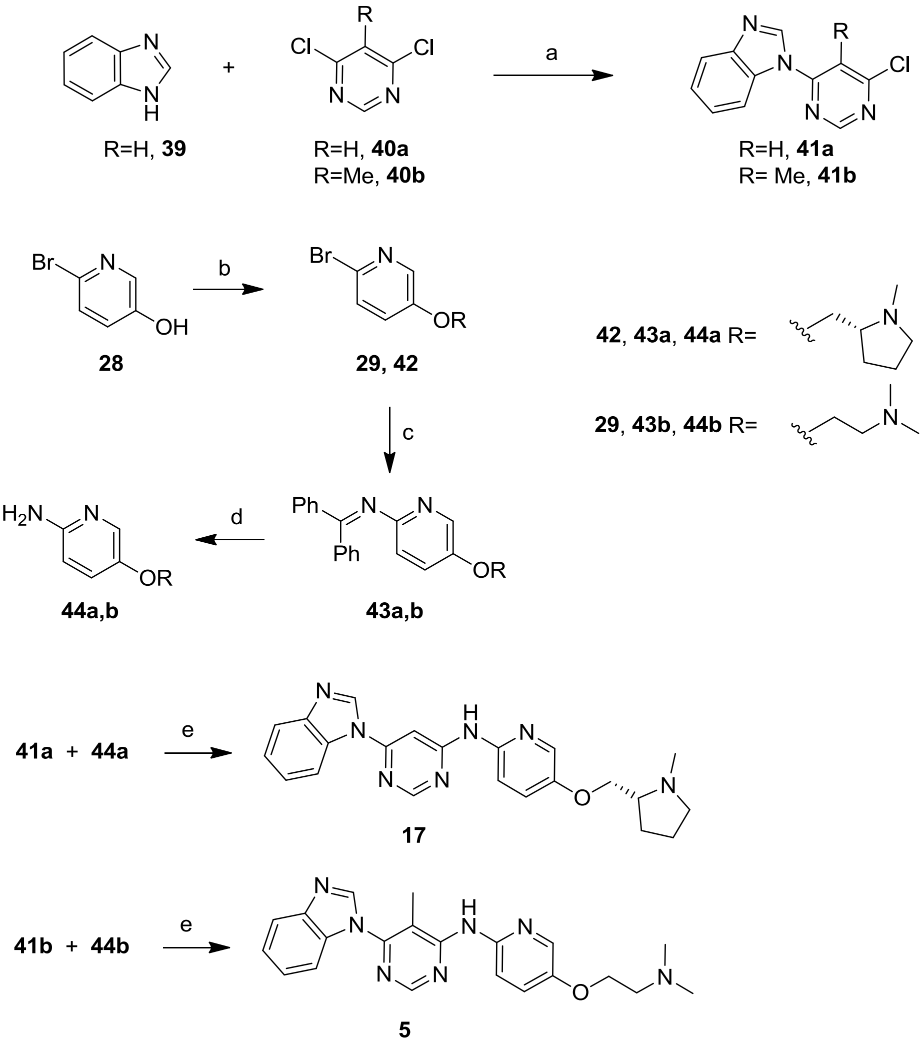

[a]Reagents and conditions: (a) Cs_2CO_3, DMF, rt, 79-82%; (b) *(R)*-(1-methylpyrrolidin-2-yl)methanol, DIAD, PPh_3, THF, 0 °C to rt, 24%; (c) benzophenone imine, $Pd(OAc)_2$, Xantphos, Cs_2CO_3, dioxane, 100 °C, 57%; (d) 2 M HCl, THF, rt, 99%; (e) Pd_2dba_3, Xantphos, NaO^tBu, toluene, 100 °C, 14-24%.

Reaction of benzimidazole (**39**) with 2,4-dichloropyrimidine (**40a/b**) using Cs_2CO_3 in DMF provided the left-hand side chloropyrimidine intermediate (**41a/b**). The right-hand side coupling partner was synthesized by first alkylating 2-bromo-5-hydroxypyridine (**28**) with *(R)*-(1-methylpyrrolidin-2-yl)methanol, conversion of the bromine into nitrogen using a palladium-catalyzed reaction with benzophenone imine followed by acid hydrolysis, to give compound **44a/b**. The chloropyrimidine and aniline were then coupled using palladium-catalysis to afford the desired product **5** in 24% and **17** in 14% yield.

Scheme 6. Synthesis of Compounds 23 and 24.[a] Combination Compounds.

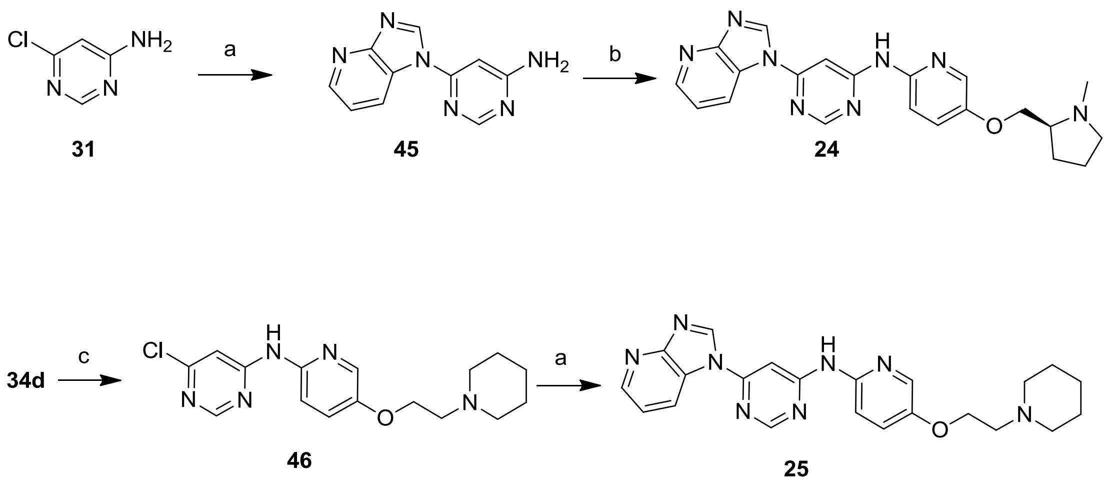

[a]Reagents and conditions: (a) 4-azabenzimidazole, NaH, DMF, 100 C, 28-43%; (b) **34b**, Pd_2dba_3, Xantphos, NaO^tBu, toluene/DMF, 100 °C, 10%; (c) **31**, Pd_2dba_3, Xantphos, NaO^tBu, toluene/DMF, 100 °C, 30%.

The synthesis of the 'combination' compounds having the favoured left and right hand sides is outlined in Scheme 6. For compound **24** the S_NAR reaction between 4-azabenzimidazole and 4-amino-6-chloropyrimidine produced 2 regioisomeric products, with the desired 4-azabenzimidazole derivative **45** produced as the major isomer over the 7-azabenzimidazole analogue. The palladium-catalyzed amination reaction with the appropriate aniline afforded the desired product in a relatively low yield. A slightly different approach was used for the synthesis of compound **25**, where the palladium-catalyzed amination of the bromopyridine **34d** with 4-amino-6-chloropyrimidine afforded compound **46**, followed by the S_NAR reaction with 4-azabenzimidazole to produce compound **25**, again with the desired 4-azabenzimidazole derivative as the major isomer, isolated in a yield of 43%.

General Synthetic Chemistry

Starting materials and solvents were purchased from commercial suppliers and were used without further purification. The petroleum ether (PE) used had a boiling point range of 60-80 °C. Microwave reactions were carried out in either a Biotage Initiator 60 or CEM Discover microwave reactor. Thin layer chromatography (TLC) analysis was performed using Merck silica gel 60 F_{254} thin layer plates. Flash silica chromatography was performed using VWR silica gel (40-63 µM), Biotage pre-packed cartridges (40-63 or 30-90 µM), Silicycle silica gel cartridges (230-400 mesh), or Grace Resolv silica cartridges. ^{1}H and ^{13}C NMR spectra were recorded on one of three instruments: 1. Bruker AMX500 instrument at 500/126 MHz; 2. Bruker Av400 at 400/100 MHz; 3. Bruker DRX400 spectrometer at 400/100 MHz. Chemical shifts (δ) are referenced to the solvent in which they were measured. Combined HPLC-MS analyses were recorded using one of three instruments: 1. Waters Alliance 2795 separations module with a Waters 2487 dual wavelength absorbance detector coupled to a Waters/Micromass LCt time of flight mass spectrometer with ESI source, detection at 254 nm; 2. Agilent 1200 series HPLC and diode array detector coupled to an Agilent 6210 time of flight (ToF) mass spectrometer (HRMS) with dual multimode APCI/ESI source, detection at 254 nm; 3. Waters 2790 LC with a 996 PDA and 2000 amu ZQ Single Quadrupole Mass Spectrometer using a Phenomenex Gemini 50 x 2.1 mm C18 column, detection at 254 nm. For LCMS instruments 1 and 2 above, analytical separations were carried out at 30 °C on a Merck Purospher STAR column (RP-18e,

30 x 4 mm) using a flow rate of 1.5 mL. The mobile phase was a mixture of methanol (solvent A) and water (solvent B), both containing formic acid at 0.1%. Gradient elution was as follows: 1:9 (A/B) to 9:1 (A/B) over 2.5 min, 9:1 (A/B) for 1 min. For LCMS instrument 3 above, separations were carried out using decreasingly polar mixtures of either water (containing 1% NH_3) and acetonitrile, or water (containing 0.1% formic acid) and acetonitrile, as eluents. All tested compounds gave >95% purity as determined by these methods, unless otherwise specified.

Experimental procedures and compound characterization

2-(4-((6-(1*H*-Benzo[d]imidazol-1-yl)pyrimidin-4-yl)amino)phenyl)ethanol (**1**)

A solution of 1-(6-chloropyrimidin-4-yl)-1H-benzo[d]imidazole (**41a**) (35 mg, 0.15 mmol) in BuOH/DMA (1:1, 0.5mL) was added to a solution of 2-(4-aminophenyl)ethanol (21 mg, 0.15 mmol) in BuOH/DMA (1:1, 0.5mL). The reaction mixture was heated to 75 °C for 24 h and then concentrated. The residue was purified by preparative HPLC (Waters XBridge Prep C18 OBD column, 5µ silica, 19 mm diameter, 100 mm length), using decreasingly polar mixtures of water (containing 0.1% NH_3) and MeCN as eluents. Fractions containing the target compound were evaporated to dryness to afford the title compound **1** (3.6 mg, 7%). ^{1}H NMR (400 MHz, DMSO) δ 9.82 (s, 1H), 8.91 (s, 1H), 8.67 (s, 1H), 8.20 (d, 1H), 7.83 – 7.76 (m, 1H), 7.59 (d, 2H), 7.49-7.35 (m, 2H), 7.22 (d, 2H), 7.13 (d, 1H), 4.59 (br s, 1H), 3.62 (t, 2H), 2.72 (t, 2H).^{13}C NMR (101 MHz, DMSO) δ 161.83, 158.59, 154.71, 144.44, 141.72, 137.11, 134.34, 131.48, 129.23, 124.14, 123.38, 120.33, 120.17, 113.71, 93.57, 62.22, 38.46. HRMS (ESI$^+$): calcd for $C_{19}H_{18}N_5O$ $(M+H)^+$: 332.1506; found: 332.1504.

6-(1*H*-Benzo[d]imidazol-1-yl)-*N*-(4-(2-(dimethylamino)ethoxy)phenyl)pyrimidin-4-amine (**2**)

In a round-bottom flask 4,6-dichloropyrimidine (0.223 g, 1.5 mmol, 1.3 eq.) and 4-(2-(dimethylamino)ethoxy)aniline **47** (0.21 g, 1.2 mmol) were dissolved in a mixture of acetic acid (5 mL) and water (1 mL) and stirred at rt overnight. The mixture was diluted with CH_2Cl_2 (20 mL), and saturated $NaHCO_3$ (aq.) was added to neutralise the aqueous layer. After gas evolution had ceased, the layers were separated and the aqueous layer was extracted with CH_2Cl_2 (2 × 20 mL). The combined organic layers were washed with brine (50 mL), dried over Na_2SO_4, filtered and concentrated. Purification by silica column chromatography (7% 1M NH_3/MeOH in CH_2Cl_2) gave the desired product 6-chloro-*N*-(4-(2-(dimethylamino)ethoxy)phenyl)pyrimidin-4-amine (**48**) as a colourless oil (0.075 g, 17% yield). ^{1}H-NMR (500 MHz, methanol-d_4) δ 8.30 (s, 1H), 7.46-7.35 (m, 2H), 7.00-6.93 (m, 2H), 6.62 (s, 1H), 4.11 (t, J = 5.4 Hz, 2H), 2.80-2.75 (m, 2H), 2.35 (d, J = 2.1 Hz, 6H). LRMS (ESI$^+$): 293 $(M+H)^+$.

To a flask was added sodium hydride (60% dispersion in mineral oil, 3.2 mg, 0.14 mmol), which was suspended in anhydrous DMF (0.5 mL). The suspension was cooled to 0 °C and benzimidazole (15 mg, 0.13 mmol) was added in one portion (gas development was observed at this stage). The mixture was stirred at 0°C until it became clear (approx. 5 mins), and **48** (34 mg, 0.12 mmol) was added. The reaction mixture was allowed to warm to rt and stirred overnight. A further equivalent of both sodium hydride and benzimidazole was added, and the reaction mixture was heated to 100 °C for an additional 7 h, cooled to rt and diluted with EtOAc (10 mL). The organic layer was washed with water (10 mL). The aqueous layer was back-extracted with EtOAc (2 × 7 mL). The combined organic layers were washed with water (3 × 25 mL), brine (25 mL), and dried over $MgSO_4$, filtered and concentrated. The residue was purified *via* HPLC (MeOH/H_2O 15 min gradient) followed by silica gel column chromatography (12% EtOH in CH_2Cl_2) to afford the title compound **2** (7 mg, 16%). ^{1}H NMR (500 MHz, MeOD) δ 8.81 (s, 1H), 8.56 (d, J = 1.0 Hz, 1H), 8.13 (d, J = 8.2 Hz, 1H), 7.76 (d, J

= 7.6 Hz, 1H), 7.54-7.48 (m, 2H), 7.45-7.36 (m, 2H), 7.03-6.99 (m, 2H), 6.94 (s, 1H), 4.17 (t, *J* = 5.4 Hz, 2H), 2.92 (t, *J* = 5.3 Hz, 2H), 2.47 (s, 6H). ^{13}C NMR (126 MHz, DMSO) δ 164.3, 160.0, 157.1, 156.6, 145.2 142.8, 133.4, 133.0, 125.8, 125.0, 124.8, 120.8, 116.2, 114.8, 94.2, 66.4, 58.9, 45.5. HRMS (ESI$^+$): calcd for $C_{21}H_{23}N_6O$ (M+H)$^+$: 375.1928; found: 375.1923.

6-(1*H*-Benzo[*d*]imidazol-1-yl)-*N*-(5-(2-(dimethylamino)ethoxy)pyridin-2-yl)pyrimidin-4-amine (**3**)
Benzimidazole (0.958 g, 8.11 mmol) was added portion wise to a suspension of NaH (60% in mineral oil, 0.357 g, 8.92 mmol) in anhydrous DMF (15 mL) at 0 °C. The reaction mixture was stirred for 20 min at 0 °C, then warmed to rt and stirred for 25 min before 4-amino-6-chloropyrimidine (**31**) (1.00 g, 7.72 mmol) was added. The reaction mixture was then heated to 100 °C for 21 h, cooled to rt, diluted with water and the resulting precipitate isolated by filtration, and washed with water to afford 6-(1*H*-benzo[*d*]imidazol-1-yl)pyrimidin-4-amine (**32**) as a light tan coloured solid (1.278 g, 78%). ^{1}H NMR (500 MHz, DMSO) δ 8.88 (s, 1H), 8.44 (d, *J* = 0.9 Hz, 1H), 8.18 (d, *J* = 8.0 Hz, 1H), 7.77 (d, *J* = 7.8 Hz, 1H), 7.43 – 7.38 (m, 1H), 7.37 – 7.32 (m, 1H), 7.24 (s, 2H), 6.81 (d, *J* = 0.9 Hz, 1H). HRMS (ESI$^+$): calcd for $C_{11}H_{10}N_5$ (M + H)$^+$, 212.0931; found 212.0937.
2-Dimethylaminoethyl chloride hydrochloride (2.51 g, 17.4 mmol) was added to a suspension of 2-bromo-5-hydroxypyridine **28** (1.52 g, 8.72 mmol) and cesium carbonate (11.36 g, 34.86 mmol) in acetone (45 mL), and the reaction mixture was heated at reflux for 15 h. After cooling to rt, the solids were removed by filtration, and the filtrate concentrated, redissolved in EtOAc, washed with 1 M NaOH (3 x), brine (2 x), dried over $MgSO_4$, filtered and concentrated. The residue was purified by silica gel column chromatography using a gradient of 1 to 7% 2M NH_3/MeOH in CH_2Cl_2 to afford 2-((6-bromopyridin-3-yl)oxy)-*N*,*N*-dimethylethanamine (**29**) as a pale yellow liquid (2.024 g, 95%). ^{1}H NMR (500 MHz, $CDCl_3$) δ 8.08 (d, *J* = 3.1 Hz, 1H), 7.36 (d, *J* = 8.6 Hz, 1H), 7.12 (dd, *J* = 8.6, 3.1 Hz, 1H), 4.08 (t, *J* = 5.6 Hz, 2H), 2.73 (t, *J* = 5.6 Hz, 2H), 2.33 (s, 6H). HRMS (ESI$^+$): calcd for $C_9H_{14}{}^{79}BrN_2O$ (M + H)$^+$, 245.0284; found 245.0286.
A mixture of 6-(1*H*-Benzo[*d*]imidazol-1-yl)pyrimidin-4-amine **32** (20 mg, 0.094 mmol), **29** (23 mg, 0.094 mmol), tris(dibenzylidineacetone)dipalladium(0) (2.6 mg, 0.0028 mmol), 4,5-bis(diphenylphosphino)-9,9-dimethylxanthene (Xantphos) (3.3 mg, 0.0056 mmol), and Cs_2CO_3 in anhydrous dioxane (1.0 mL) was degassed and backfilled with argon (3 x). The flask was heated to 100 °C in a sealed flask for 18 h, cooled to rt, diluted with EtOAc, washed successively with water (1 x), brine (1 x), dried over $MgSO_4$, filtered, and concentrated. The residue was purified by silica column chromatography using a gradient of 2.5% to 15% MeOH in CH_2Cl_2 to afford the title compound **3** as a white solid (20 mg, 57%). ^{1}H NMR (500 MHz, $CDCl_3$) δ 8.76 (s, 1H), 8.71 (d, *J* = 0.9 Hz, 1H), 8.28 (s, 1H), 8.21 (d, *J* = 8.0 Hz, 1H), 8.12 (d, *J* = 3.0 Hz, 1H), 7.88 (d, *J* = 7.7 Hz, 1H), 7.56 (s, 1H), 7.46 – 7.41 (m, 1H), 7.41 – 7.37 (m, 1H), 7.34 (dd, *J* = 8.9, 3.0 Hz, 1H), 7.17 (d, *J* = 8.8 Hz, 1H), 4.13 (t, *J* = 5.6 Hz, 2H), 2.76 (t, *J* = 5.6 Hz, 2H), 2.37 (s, 6H). ^{13}C NMR (126 MHz, DMSO) δ 160.72, 158.32, 155.14, 150.63, 146.62, 144.54, 141.64, 134.07, 131.43, 125.02, 124.37, 123.44, 120.25, 114.31, 113.61, 94.23, 66.58, 57.62, 45.44. HRMS (ESI$^+$): calcd for $C_{20}H_{22}N_7O$ (M + H)$^+$, 376.1880; found 376.1862.

6-(1*H*-Benzo[d]imidazol-1-yl)-*N*-(5-(3-(dimethylamino)propoxy)pyridin-2-yl)pyrimidin-4-amine (**4**)
A mixture of 2-bromo-5-hydroxypyridine **28** (0.200 g, 1.15 mmol), 2-dimethylamino-1-propyl chloride hydrochloride (0.200 g, 1.64 mmol), and K_2CO_3 (0.477 g, 3.45 mmol) in anhydrous DMF (4.0 mL) was heated to 70 °C for 20 h, cooled to rt, diluted with water and saturated $NaHCO_3$ (aq.), extracted with EtOAc (3 x). The organic phase was washed with brine (1 x), dried over $MgSO_4$, filtered and concentrated. Heptane was added and concentrated (2 x) to remove residual DMF. The residue was purified by silica column chromatography using a gradient of 2% to 5% MeOH in CH_2Cl_2, then to 3% 2M NH_3/MeOH in CH_2Cl_2 to afford 3-((6-bromopyridin-3-yl)oxy)-*N*,*N*-

dimethylpropan-1-amine (**34a**) as a pale yellow oil (165 mg, 55%). ^{1}H NMR (500 MHz, $CDCl_3$) δ 8.06 (d, *J* = 3.1 Hz, 1H), 7.35 (d, *J* = 8.5 Hz, 1H), 7.10 (dd, *J* = 8.7, 3.2 Hz, 1H), 4.05 (t, *J* = 6.4 Hz, 2H), 2.44 (t, *J* = 7.1 Hz, 2H), 2.25 (s, 6H), 1.96 (p, *J* = 6.6 Hz, 2H). HRMS (ESI$^+$): calcd for $C_{10}H_{16}{}^{79}BrN_2O$ $(M + H)^+$, 259.0440; found 259.0452.

A flask was charged with **32** (48 mg, 0.23 mmol), **34a** (59 mg, 0.23 mmol), tris(dibenzylideneacetone)dipalladium(0) (6 mg, 0.007 mmol), Xantphos (8 mg, 0.01 mmol), and cesium carbonate (0.111 g, 0.342 mmol) in anhydrous dioxane (2.0 mL), then evacuated and backfilled with argon (4 x), sealed, and heated to 100 °C for 28 h, cooled to rt and added additional tris(dibenzylideneacetone)dipalladium(0) (6 mg, 0.007 mmol), Xantphos (8 mg, 0.01 mmol), and cesium carbonate (0.111 g, 0.342 mmol). The flask was evacuated and backfilled with argon (3 x), sealed, and heated to 100 °C for 19 h, cooled to rt, diluted with EtOAc, washed with water (1 x). The aqueous phase was extracted with EtOAc (2 x), and the combined organic phases were washed with brine (1 x), dried over $MgSO_4$, filtered and concentrated. The residue was purified by silica column chromatography using a gradient of 2.5% to 10% MeOH in CH_2Cl_2 to afford the title compound **4** as an off-white solid (51 mg, 58%). ^{1}H NMR (500 MHz, $CDCl_3$) δ 8.76 (s, 1H), 8.70 (d, *J* = 0.9 Hz, 1H), 8.26 (br s, 1H), 8.21 (d, *J* = 8.1 Hz, 1H), 8.10 (d, *J* = 2.9 Hz, 1H), 7.88 (d, *J* = 7.5 Hz, 1H), 7.70 (br s, 1H), 7.46 – 7.41 (m, 1H), 7.41-7.37 (m, 1H), 7.31 (dd, *J* = 8.9, 3.0 Hz, 1H), 7.18 (br d, *J* = 8.8 Hz, 1H), 4.09 (t, *J* = 6.4 Hz, 2H), 2.50 (t, *J* = 7.1 Hz, 2H), 2.29 (s, 6H), 2.05 – 1.95 (m, 2H). ^{13}C NMR (126 MHz, $CDCl_3$) δ 160.68, 158.69, 156.50, 151.70, 146.26, 145.27, 141.36, 134.58, 132.00, 125.45, 124.79, 123.91, 121.07, 113.97, 113.65, 94.52, 67.30, 56.26, 45.52, 27.51. HRMS (ESI$^+$): calcd for $C_{21}H_{24}N_7O$ $(M + H)^+$, 390.2037; found 390.2041.

6-(1*H*-Benzo[*d*]imidazol-1-yl)-*N*-(5-(3-(dimethylamino)propoxy)pyridin-2-yl)-5-methylpyrimidin-4-amine (**5**)

Benzimidazole (161 mg, 1.36 mmol) was added to 4,6-dichloro-5-methylpyrimidine (444 mg, 2.72 mmol) and cesium carbonate (887 mg, 2.72 mmol) in DMF (10 mL) at 20°C under nitrogen. The resulting suspension was stirred at 20 °C for 2 hours. The reaction mixture was filtered to remove insolubles. The filtrate was concentrated *in vacuo*. The residue was dissolved in CH_2Cl_2 (100 mL) and washed with water (100 mL). The organic layer was passed through a phase separating cartridge and concentrated under reduced pressure to give a beige solid. The crude product was purified by flash silica chromatography, elution gradient 0 to 50% EtOAc in isohexane. Pure fractions were evaporated to dryness to afford compound 1-(6-chloro-5-methylpyrimidin-4-yl)-1*H*-benzo[*d*]imidazole **41b** as a white solid (255 mg, 77%). ^{1}H NMR (400 MHz, $CDCl_3$) δ 8.89 (s, 1H), 8.23 (s, 1H), 7.96 – 7.78 (m, 1H), 7.59 – 7.49 (m, 1H), 7.46 – 7.34 (m, 2H), 2.51 – 2.40 (m, 3H). LRMS (ESI$^+$): 245 $(M+H)^+$.

To a stirring solution of **29** (1.00 g, 4.08 mmol, 1.0 eq.) in anhydrous dioxane (10 ml) under argon was added benzophenone imine (0.74 g, 4.1 mmol, 1.0 eq.), cesium carbonate (2.66 g, 8.61 mmol, 2.0 equiv.), Xantphos (0.47 g, 0.82 mmol, 0.2 eq.) and palladium(II) acetate (92 mg, 0.41 mmol, 0.1 equiv.). The reaction mixture was evacuated and backfilled with argon (4 x) and then heated to 100 °C for 4.5 h. The reaction mixture was diluted with CH_2Cl_2 (100 ml) and the solids filtered off. The filtrate was washed with water (50 ml), brine (50 ml), dried (Na_2SO_4) and the resulting residue dry-loaded onto silica, followed by purification by silica column chromatography (0-20% MeOH/ CH_2Cl_2) to afford 5-(2-(dimethylamino)ethoxy)-*N*-(diphenylmethylene)pyridin-2-amine (**43b**) (85%) as a mixture with unreacted starting material (12%) as a yellow oil. ^{1}H NMR (500 MHz, $CDCl_3$) δ 8.02 (d, *J* = 3.0 Hz, 1H), 7.83 – 7.76 (m, 2H), 7.48 (t, *J* = 7.4 Hz, 1H), 7.41 (t, *J* = 7.6 Hz, 2H), 7.33 – 7.22 (m, 3H), 7.15 (dd, *J* = 7.9, 1.5 Hz, 2H), 7.05 (dd, *J* = 8.7, 3.0 Hz, 1H), 6.53 (d, *J* = 8.8 Hz, 1H), 4.04 (t, *J* = 5.6 Hz, 2H), 2.72 (t, *J* = 5.6 Hz, 2H), 2.35 (s, 6H). LRMS (ESI$^+$): 346 $(M + H)^+$.

2M HCl (aq.) (21.71 mL, 43.42 mmol) was added to a stirred solution of **43b** (6.00 g, 17.4 mmol) in THF (100 mL) and then stirred at rt for 16 h. The reaction mixture was diluted with MeOH and

purified by ion exchange chromatography, using an SCX-2 column. The desired product was eluted from the column using 7M NH_3/MeOH and pure fractions were evaporated to dryness to afford compound 5-(2-(dimethylamino)ethoxy)pyridin-2-amine (**44b**) as a brown oil which solidified on standing (2.70 g, 86%). ^{1}H NMR (400 MHz, $CDCl_3$) δ 7.78 (d, *J* = 2.8 Hz, 1H), 7.11 (dd, *J* = 3.0, 8.8 Hz, 1H), 6.53 – 6.37 (m, 1H), 4.13 (s, 2H), 4.00 (t, *J* = 5.6 Hz, 2H), 2.69 (t, *J* = 5.6 Hz, 2H), 2.32 (s, 6H). LRMS (ESI$^+$): 182 $(M + H)^+$.

Tris(dibenzylideneacetone)dipalladium(0) (46.8 mg, 0.05 mmol) was added to **44b** (200 mg, 1.02 mmol), **41b** (250 mg, 1.02 mmol), sodium *t*-butoxide (147 mg, 1.53 mmol) and Xantphos (89 mg, 0.15 mmol) in toluene (10 mL) at 20°C under nitrogen. The resulting suspension was stirred at 100 °C for 16 hours then cooled to room temperature. The suspension was filtered and concentrated *in vacuo*. The residue was dissolved in CH_2Cl_2 (100 mL), washed with water (50 mL) and the organic layer was passed through a phase separating cartridge and concentrated *in vacuo* to give a brown solid. The crude product was purified by preparative HPLC (Waters XBridge Prep C18 OBD column, 5μ, 19 mm x 100 mm), using decreasingly polar mixtures of water (containing 1% NH_3) and MeCN as eluents. Fractions containing the desired compound were evaporated to dryness to afford the title compound **5** as a brown solid (99 mg, 24%). ^{1}H NMR (400 MHz, DMSO-d_6, 20 °C) δ 9.28 (s, 1H), 8.58 (s, 2H), 8.11 (d, *J* = 3.0 Hz, 1H), 8.00 (d, *J* = 9.0 Hz, 1H), 7.74 - 7.83 (1H, m), 7.45 - 7.59 (2H, m), 7.28 - 7.39 (2H, m), 4.03 - 4.15 (m, 2H), 2.38 (m, 2H), 2.16 (s, 9H), d 1.80 - 1.95 (m, 2H). ^{13}C NMR (126 MHz, DMSO-d_6, 30°C) δ 160.79, 155.22, 152.77, 151.71, 145.53, 143.37, 143.07, 134.63, 133.02, 123.64, 123.54, 122.65, 119.78, 117.29, 112.18, 108.86, 66.63, 55.49, 45.12, 26.84, 11.98. HRMS (ESI$^+$): calcd for $C_{21}H_{26}N_7O$ $(M + H)^+$, 404.2193; found 404.2193.

N-(5-(2-(Dimethylamino)ethoxy)pyridin-2-yl)-6-(1*H*-imidazol-1-yl)pyrimidin-4-amine (**6**)

A flask was charged with **29** (0.886 g, 3.61 mmol), cesium carbonate (1.65 g, 5.06 mmol), Xantphos (0.138 g, 0.239 mmol), palladium(II) acetate (49 mg, 0.22 mmol), and evacuated and backfilled with argon (3 x). Dioxane (17 mL) was added, and the flask was evacuated and backfilled with argon (3 x) again. The reaction mixture was heated to 70 °C for 15 min, then 4-amino-6-chloropyrimidine (0.562 g, 4.34 mmol) was added, and the reaction mixture was heated at reflux for 17 h, after which time additional quantities of cesium carbonate (1.65 g, 5.06 mmol), Xantphos (0.138 g, 0.239 mmol), and palladium(II) acetate (49 mg, 0.22 mmol) were added, the flask was evacuated and backfilled with argon (3 x) and heated at reflux for 5.5 h. The reaction mixture was cooled to rt, diluted with EtOAc, washed with water (1 x). The aqueous phase was filtered to remove a brown precipitate and extracted with EtOAc (1 x). The combined organic layers were washed with brine (1 x), dried over $MgSO_4$, filtered and concentrated. The residue was purified purified by silica column chromatography using a gradient of 3 to 12% MeOH in CH_2Cl_2 to afford 6-chloro-*N*-(5-(2-(dimethylamino)ethoxy)pyridin-2-yl)pyrimidin-4-amine (**30**) as a pale yellow solid (0.508 g, 43%). ^{1}H NMR (500 MHz, $CDCl_3$) δ 8.52 (d, *J* = 0.8 Hz, 1H), 8.06 (ap t, *J* = 1.8 Hz, 1H), 7.66 (br s, 1H), 7.48 (br s, 1H), 7.31 (d, *J* = 1.7 Hz, 2H), 4.11 (t, *J* = 5.6 Hz, 2H), 2.74 (t, *J* = 5.6 Hz, 2H), 2.35 (s, 6H). HRMS (ESI$^+$): calcd for $C_{13}H_{17}{}^{35}ClN_5O$ $(M + H)^+$, 294.1116; found 294.1121.

Imidazole (21 mg, 0.31 mmol) was added to a suspension of sodium hydride (60% in mineral oil, 13 mg, 0.34 mmol) in anhydrous DMF (0.75 mL) at 0 °C. The reaction mixture was stirred at 0 °C for 10 min, then warmed to rt for 25 min before **30** (75 mg, 0.26 mmol) was added. The reaction mixture was heated to 100 °C for 22 h, cooled to rt, diluted with water, extracted with EtOAc (2x). The organic phase was washed with brine (1 x). The combined aqueous phases were then extracted with CH_2Cl_2 (1 x), and the combined organic phases were dried over Na_2SO_4, filtered, and concentrated. Heptane was added and the mixture concentrated (3 x) to remove residual DMF. The residue was purified by silica column chromatography using a gradient of 3% to 13% MeOH in CH_2Cl_2 to afford the title compound **6** as a white solid (50 mg, 60%). ^{1}H NMR (500 MHz, $CDCl_3$) δ 8.60 (d, *J* = 0.9

Hz, 1H), 8.46 – 8.41 (m, 1H), 8.09 (d, *J* = 3.0 Hz, 1H), 7.85 (s, 1H), 7.71 (s, 1H), 7.67 (m, 1H), 7.32 (dd, *J* = 8.9, 3.0 Hz, 1H), 7.24 – 7.18 (m, 2H), 4.12 (t, *J* = 5.6 Hz, 2H), 2.76 (t, *J* = 5.6 Hz, 2H), 2.36 (s, 6H). ^{13}C NMR (126 MHz, $CDCl_3$) δ 160.81, 158.65, 155.61, 151.64, 146.27, 135.46, 134.48, 131.26, 125.70, 116.02, 114.07, 93.07, 67.25, 58.45, 46.07. HRMS (ESI$^+$): calcd for $C_{16}H_{20}N_7O$ (M + H)$^+$, 326.1724; found 326.1733.

N-(5-(2-(Dimethylamino)ethoxy)pyridin-2-yl)-6-(1*H*-indazol-1-yl)pyrimidin-4-amine (**7**)

To a suspension of **30** (50 mg, 0.17 mmol) and indazole (30 mg, 0.26 mmol) in anhydrous toluene (1.7 mL) was added NaO^{t}Bu (25 mg, 0.26 mmol), Tris(dibenzylideneacetone)dipalladium(0) (3.1 mg, 0.0034 mmol), and Xantphos (5.9 mg, 0.0010 mmol). The flask was evacuated and backfilled with argon (3 x), and the reaction mixture was heated to 100 °C for 7 h. The reaction mixture was cooled to rt, and additional NaO^{t}Bu (25 mg, 0.26 mmol), Tris(dibenzylideneacetone)dipalladium(0) (3.1 mg, 0.0034 mmol), and Xantphos (5.9 mg, 0.0010 mmol) were added, the flask was evacuated and backfilled with argon (3 x), and the reaction mixture was heated to 100 °C for 23 h. The reaction mixture was again cooled to rt, to which anhydrous dioxane (1.0 mL) and additional Tris(dibenzylideneacetone)dipalladium(0) (16 mg, 0.017 mmol), and Xantphos (30 mg, 0.052 mmol) were added, the flask was evacuated and backfilled with argon (3 x), and the reaction mixture was heated to 100 °C for 23 h, then at 85 °C for 3 days, cooled to rt, diluted with EtOAc, washed with water (1 x). The aqueous phase was extracted with EtOAc (1 x) and the combined organic phases were washed with brine (1 x), dried over Na_2SO_4, filtered and concentrated. Attempts to purify by silica column chromatography (2.5% to 8% MeOH in CH_2Cl_2) were unsuccessful, providing a 30 mg mixture of the N1 and N2 substituted indazole compounds. The residue was then purified by HPLC to afford the title compound **7** as a white solid (2.9 mg, 4.5%). HPLC conditions: Gilson GX-281 with Phenomenex Gemini column (250 x 10 mm), elution at 5 mL/min with 20% to 40% MeOH + 0.1% formic acid in water + 0.1% formic acid over 30 min. ^{1}H NMR (500 MHz, MeOD) δ 8.85 (d, *J* = 9.2 Hz, 1H), 8.65 (s, 1H), 8.32 (s, 1H), 8.21-8.13 (m, 2H), 7.84 (d, *J* = 8.0 Hz, 1H), 7.61 (d, *J* = 8.0 Hz, 1H), 7.56 (ddd, *J* = 8.3, 7.0, 1.1 Hz, 1H), 7.51-7.47 (m, 1H), 7.35-7.32 (m, 1H), 4.30 (t, *J* = 5.2 Hz, 2H), 3.16 (t, *J* = 5.2 Hz, 2H), 2.65 (s, 6H).^{13}C NMR (126 MHz, MeOD) δ 160.72, 159.91, 157.43, 150.67, 147.53, 139.13, 138.34, 134.13, 128.02, 126.41, 125.08, 122.86, 120.71, 115.51, 114.64, 92.55, 64.45, 56.99, 43.41. LRMS (ESI$^+$): 376.2 (M + H)$^+$.

N-(5-(2-(Dimethylamino)ethoxy)pyridin-2-yl)-6-(1*H*-indol-1-yl)pyrimidin-4-amine (**8**)

Indole (90 mg, 0.77 mmol) was added to a suspension of sodium hydride (60% in mineral oil, 34 mg, 0.84 mmol) in anhydrous DMF (1.25 mL) at 0 °C. An additional 1.25 ml of anhydrous DMF was added to try and dissolve the substantial amount of solid in the reaction mixture. The reaction mixture was stirred at 0 °C for 20 min, and then at rt for 15 min (solids dissolved), before **30** (75 mg, 0.26 mmol) was added and the reaction mixture was heated to 100 °C for 7.5 h, then at 80 °C for 15 h. The reaction mixture was cooled to rt, diluted with water, extracted with EtOAc (2 x). The organic phases were washed with water (1 x), brine (1 x), dried over Na_2SO_4, filtered and concentrated. Heptane was added and concentrated (2 x) to remove residual DMF. Purification by silica column chromatography (3% to 8.5% MeOH in CH_2Cl_2) proved unsatisfactory, and the resulting material was purified by HPLC (Gilson GX-281 with Phenomenex Gemini column (250 x 10 mm), elution at 5 mL/min with 35% MeOH + 0.1% formic acid, 65% water + 0.1% formic acid for 4 min, then increased to 40% MeOH + 1% formic acid over 6 min, and then to 80% MeOH + 0.1% formic acid over 1.5 min and held for 1 min) to afford the title compound **8** as an off-white solid (8 mg, 8%). ^{1}H NMR (500 MHz, MeOD) δ 8.60 (br s, 1H), 8.36 (d, *J* = 8.4 Hz, 1H), 8.14 (br s, 1H), 7.98 (br s, 1H), 7.88 (d, *J* = 3.5 Hz, 1H), 7.62 (d, *J* = 7.8 Hz, 1H), 7.56 (br d, *J* = 8.5 Hz, 1H), 7.48 (br d, *J* = 8.4 Hz, 1H), 7.32-7.27 (m, 1H), 7.22-7.18 (m, 1H), 6.74 (d, *J* = 3.5 Hz, 1H), 4.41 – 4.34 (m, 2H), 3.52 – 3.42 (m, 2H), 2.90 (s,

6H). ^{13}C NMR (126 MHz, MeOD) δ 162.16, 159.45, 159.20, 151.58, 149.33, 136.50, 135.45, 132.58, 126.62, 126.36, 124.57, 123.02, 122.19, 115.76, 115.25, 107.91, 95.24, 64.73, 57.89, 44.17. HRMS (ESI$^+$): calcd for $C_{21}H_{23}N_6O$ (M + H)$^+$, 375.1928; found 375.1920.

N-(5-(2-(Dimethylamino)ethoxy)pyridin-2-yl)-6-(quinolin-3-yl)pyrimidin-4-amine (**9**)

A mixture of quinoline-3-boronic acid (41 mg, 0.24 mmol), **30** (50 mg, 0.17 mmol), $Pd_2(PPh_3)_2Cl_2$ (6 mg, 0.008 mmol), tetrabutylammonium bromide (5 mg, 0.02 mmol), and K_2CO_3 (59 mg, 0.43 mmol) in 3/1 dioxane/water (2.0 mL) was heated to 90 °C for 4 h. The reaction mixture was cooled to rt, concentrated, diluted with EtOAc, washed with saturated $NaHCO_3$ (aq.) (1 x), brine (1 x), dried over Na_2SO_4, filtered and concentrated. The residue was purified by silica column chromatography using a gradient of 2.5% to 7.5% 2M NH_3/MeOH in CH_2Cl_2 to afford the title compound **9** as a pale yellow solid (47 mg, 72%). ^{1}H NMR (500 MHz, $CDCl_3$) δ 9.55 (d, *J* = 2.2 Hz, 1H), 8.90-8.87 (m, 2H), 8.17 (d, *J* = 8.1 Hz, 1H), 8.14 – 8.07 (m, 2H), 7.97 (dd, *J* = 8.2, 1.1 Hz, 1H), 7.79 (ddd, *J* = 8.4, 6.9, 1.4 Hz, 1H), 7.64-7.58 (m, 2H), 7.51 (br d, *J* = 8.9 Hz, 1H), 7.34 (dd, *J* = 8.9, 3.0 Hz, 1H), 4.13 (t, *J* = 5.6 Hz, 2H), 2.76 (t, *J* = 5.6 Hz, 2H), 2.36 (s, 6H). ^{13}C NMR (126 MHz, $CDCl_3$) δ 161.62, 160.02, 158.89, 151.59, 149.13, 149.07, 146.43, 134.93, 134.77, 130.74, 130.33, 129.55, 128.94, 127.83, 127.38, 125.45, 114.20, 103.04, 67.26, 58.48, 46.09. HRMS (ESI$^+$): calcd for $C_{22}H_{23}N_6O$ (M + H)$^+$, 387.1928; found 387.1916.

N-(5-(2-(Dimethylamino)ethoxy)pyridin-2-yl)-6-(3*H*-imidazo[4,5-*b*]pyridin-3-yl)pyrimidin-4-amine (**10**) and *N*-(5-(2-(dimethylamino)ethoxy)pyridin-2-yl)-6-(1*H*-imidazo[4,5-*b*]pyridin-1-yl)pyrimidin-4-amine (**11**)

4-Azabenzimidazole (32 mg, 0.266 mmol) was added to a suspension of NaH (60% in mineral oil, 12 mg, 0.29 mmol) in anhydrous DMF (0.75 mL) at 0 °C. The reaction mixture was stirred at 0 °C for 15 min, then warmed to rt for 15 min before **30** (65 mg, 0.22 mmol) was added. The reaction mixture was then heated to 100 °C for 18 h. The reaction mixture was diluted with water, and the resulting precipitate isolated by filtration, washed with water. The residue was purified by silica column chromatography using a gradient of 5% to 15% MeOH in CH_2Cl_2 to afford first compound **10** as a white solid (7 mg, 8%), followed by compound **11** as an off-white solid (36 mg, 43%).

Data for compound **10**: ^{1}H NMR (500 MHz, $CDCl_3$) δ 9.24 (s, 1H), 8.82 (s, 1H), 8.72 (d, *J* = 0.9 Hz, 1H), 8.52 (dd, *J* = 4.8, 1.5 Hz, 1H), 8.46 (s, 1H), 8.19 – 8.14 (m, 2H), 7.90 (d, *J* = 8.9 Hz, 1H), 7.40 – 7.34 (m, 2H), 4.14 (t, *J* = 5.6 Hz, 2H), 2.77 (t, *J* = 5.6 Hz, 2H), 2.37 (s, 6H). HRMS (ESI$^+$): calcd for $C_{19}H_{21}N_8O$ (M + H)$^+$, 377.1833; found 377.1829.

Data for compound **11**: ^{1}H NMR (500 MHz, $CDCl_3$) δ 9.00 (s, 1H), 8.73 (d, *J* = 1.0 Hz, 1H), 8.68 (dd, *J* = 4.7, 1.6 Hz, 1H), 8.65 (dd, *J* = 8.2, 1.6 Hz, 1H), 8.35 (s, 1H), 8.14 (d, *J* = 3.0 Hz, 1H), 7.65 (s, 1H), 7.39 (dd, *J* = 8.2, 4.8 Hz, 1H), 7.36 (dd, *J* = 8.9, 3.0 Hz, 1H), 7.15 (d, *J* = 8.8 Hz, 1H), 4.15 (t, *J* = 5.6 Hz, 2H), 2.78 (t, *J* = 5.6 Hz, 2H), 2.38 (s, 6H). A NOESY NMR confirmed this regioisomer *via* a correlation between H7 of the azabenzimidazole moiety and the H5 of the pyrimidine.^{13}C NMR (126 MHz, DMSO-d_6) δ 160.75, 158.34, 156.72, 154.91, 150.73, 146.50, 145.41, 143.77, 134.16, 125.02, 124.18, 122.34, 119.63, 114.38, 94.08, 66.69, 57.67, 45.50. HRMS (ESI$^+$): calcd for $C_{19}H_{21}N_8O$ (M + H)$^+$, 377.1833; found 377.1829.

N-(5-(2-(dimethylamino)ethoxy)pyridin-2-yl)-6-(6-methyl-1*H*-benzo[*d*]imidazol-1-yl)pyrimidin-4-amine (**12**)

5-Methylbenzimidazole (34 mg, 0.26 mmol) was added to a suspension of sodium hydride (60% in mineral oil, 11 mg, 0.28 mmol) in anhydrous DMF (0.75 mL) at 0 °C. The reaction mixture was stirred for 10 min at 0 °C and then at rt for a further 20 min before **30** (75 mg, 0.26 mmol) was added.

The reaction mixture was heated to 100 °C for 24 h, cooled to rt, diluted with water and the resulting precipitate was isolated by filtration, washed with water. The crude material was purified by HPLC to afford first the title compound **12** as a white solid (9 mg, 9%), and followed by the corresponding regioisomer as a white solid (18 mg, 18%). HPLC conditions: Gilson GX-281 with Phenomenex Gemini column (250 x 10 mm), elution at 5 mL/min with 20% MeOH + 0.1% formic acid, 80% water + 0.1% formic acid for 6 min, then increased to 30% MeOH + 1% formic acid over 1 min, and held for 15 min. ^{1}H NMR (500 MHz, MeOD) δ 8.80 (s, 1H), 8.65 (s, 1H), 8.55 (br s, 1H), 8.16 (s, 1H), 8.12 (d, *J* = 2.9 Hz, 1H), 8.06 (s, 1H), 7.63 (d, *J* = 8.2 Hz, 1H), 7.54 (d, *J* = 9.0 Hz, 1H), 7.46 (dd, *J* = 9.0, 3.0 Hz, 1H), 7.24 (d, *J* = 8.2 Hz, 1H), 4.24 (t, *J* = 5.3 Hz, 2H), 3.01 (t, *J* = 5.3 Hz, 2H), 2.55 (s, 3H), 2.53 (s, 6H). Note that a NOE signal between H7 of the benzimidazole moiety and the H5 of the pyrimidine confirmed this regioisomer.^{13}C NMR (126 MHz, MeOD) δ 162.52, 159.62, 157.02, 152.43, 148.52, 143.40, 142.31, 136.30, 135.42, 133.09, 126.54, 126.28, 120.41, 115.89, 114.78, 95.88, 66.67, 58.73, 45.32, 22.13. HRMS (ESI$^+$): calcd for $C_{21}H_{24}N_7O$ (M + H)$^+$, 390.2037; found 390.2038.

N-(5-(2-(dimethylamino)ethoxy)pyridin-2-yl)-6-(2-methyl-1*H*-benzo[*d*]imidazol-1-yl)pyrimidin-4-amine (**13**).

A flask was charged with 2-methyl benzimidazole (40 mg, 0.30 mmol), **30** (75 mg, 0.26 mmol), palladium(II) acetate (5 mg, 0.020 mmol), Xantphos (24 mg, 0.01 mmol), and cesium carbonate (0.116 g, 0.511 mmol) in anhydrous dioxane (1.5 mL), then evacuated and backfilled with argon (3 x), sealed, and heated to 120 °C for 2 h, cooled to rt and added additional palladium(II) acetate (5 mg, 0.020 mmol) and Xantphos (24 mg, 0.01 mmol). The flask was evacuated and backfilled with argon (3 x), sealed, and heated to 150 °C for 2 h, cooled to rt, diluted with EtOAc, washed with water (1 x). The aqueous phase was extracted with EtOAc (2 x), and the combined organic phases were washed with brine (1 x), dried over Na_2SO_4, filtered and concentrated. The residue was purified by silica column chromatography using a gradient (1% NH_3 in MeOH in 2% MeOH/ CH_2Cl_2 to 1% NH_3 in MeOH in 10% MeOH/ CH_2Cl_2) to afford the title compound **13** as an off-white solid (21 mg, 21%). ^{1}H NMR (500 MHz, MeOD) δ 8.76 (d, *J* = 1.0 Hz, 1H), 8.08 (d, *J* = 3.5 Hz, 1H), 8.04 (br s, 1H), 7.74 – 7.64 (m, 2H), 7.57 (br s, 1H), 7.48 (dd, *J* = 9.0, 3.1 Hz, 1H), 7.39 – 7.32 (m, 2H), 4.20 (t, *J* = 5.4 Hz, 2H), 2.89 (t, *J* = 5.3 Hz, 2H), 2.78 (s, 3H), 2.44 (s, 6H). ^{13}C NMR (126 MHz, MeOD) δ 161.45, 158.50, 155.65, 151.78, 151.36, 146.71, 141.72, 134.02, 133.97, 124.89, 123.36, 123.14, 118.04, 114.52, 111.00, 100.98, 65.87, 57.56, 44.24, 14.10. HRMS (ESI$^+$): calcd for $C_{21}H_{24}N_7O$ (M + H)$^+$, 390.2037; found 390.2043.

6-(1*H*-Benzo[*d*]imidazol-1-yl)-*N*-(pyridin-2-yl)pyrimidin-4-amine (**14**)

Benzimidazole (63.1 mg, 0.53 mmol) was added to a stirred suspension of NaH (21.4 mg, 0.53 mmol, 60% mineral oil dispersion) in DMA (2 mL) at rt under nitrogen. After 3 minutes, 6-chloro-*N*-(pyridin-2-yl)pyrimidin-4-amine[16] (50.2 mg, 0.24 mmol) was added and the resulting solution was stirred at 100 °C for 18 h. The reaction mixture was allowed to cool, quenched with water (10 mL) and the precipitate was collected by filtration. The solid was suspended in DMSO/MeCN/H_2O (7:2:1) (5 mL) then filtered and dried under vacuum at 60 °C to afford the title compound **14** as a solid (58 mg, 83%). ^{1}H NMR (400 MHz, DMSO-d_6) δ 10.51 (s, 1H), 8.95 (s, 1H), 8.79 (d, *J* = 0.90 Hz, 1H), 8.44 – 8.39 (m, 1H), 8.37 (s, 1H), 8.27 (d, *J* = 8.11 Hz, 1H), 7.87 – 7.76 (m, 2H), 7.70 (d, *J* = 8.31 Hz, 1H), 7.52 – 7.43 (m, 1H), 7.42 – 7.36 (m, 1H), 7.08 (ddd, *J* = 0.97, 4.95, 7.23 Hz, 1H). ^{13}C NMR (176 MHz, DMSO-d_6) δ 160.96, 158.61, 155.62, 153.16, 147.98, 144.61, 141.98, 138.49, 131.66, 124.82, 123.97, 120.50, 118.36, 113.94, 113.69, 95.42. HRMS (ESI$^+$): calcd for $C_{16}H_{13}N_6$ (M + H)$^+$, 289.1196; found, 289.1194.

6-(1*H*-Benzo[*d*]imidazol-1-yl)-*N*-(5-(3-(dimethylamino)propyl)pyridin-2-yl)pyrimidin-4-amine (**15**)

A solution of 3-(6-chloro-pyridin-3-yl)acrylic acid ethyl ester (0.500 g, 2.36 mmol) in anhydrous diethyl ether (10 mL) was added over a period of 30 min to a solution of $LiAlH_4$ (1M in diethyl ether, 15 mL, 15 mmol) at 0 °C. The reaction mixture was stirred at 0 °C for an additional 10 min, then warmed to rt for 50 min, diluted with ether (25 mL), cooled in ice bath and carefully quenched with water (0.2 mL), then 1M aq. NaOH (0.8 mL). The resulting mixture was stirred at rt for 15 min, added $MgSO_4$ and stirred for another 15 min, filtered, and concentrated. The residue was purified by silica column chromatography using 3:2 PE:EtOAc to afford compound 3-(6-chloropyridin-3-yl)propan-1-ol (**37**)[17] as a colourless oil (61 mg, 15%). ^{1}H NMR (500 MHz, $CDCl_3$) δ 8.24 (d, *J* = 2.4 Hz, 1H), 7.49 (dd, *J* = 8.2, 2.5 Hz, 1H), 7.25 (d, *J* = 8.2 Hz, 1H), 3.69 (q, *J* = 6.2 Hz, 2H), 2.74 – 2.67 (m, 2H), 1.91 – 1.84 (m, 2H), 1.30 (t, *J* = 5.0 Hz, 1H). LRMS (ESI^+) 172.06 $(M + H)^+$.

A solution of methanesulfonyl chloride (0.030 mL, 0.38 mmol) in anhydrous CH_2Cl_2 (1.0 mL) was added drop-wise to a solution of **37**[17] (60 mg, 0.35 mmol) and DIEA (0.122 mL, 0.699 mmol) in anhydrous CH_2Cl_2 (4.0 mL) at 0 °C. The reaction mixture was stirred at 0 °C for 2 h, quenched with water, and extracted with EtOAc (3 x). The organic phase was washed with brine (1 x), dried over $MgSO_4$, filtered and concentrated. The crude intermediate was dissolved in anhydrous THF (0.5 mL) and cooled to 0 °C. To this was added dimethylamine (2M in THF, 0.524 mL, 1.05 mmol) drop-wise. The reaction mixture was allowed to warm to rt and stirred for 15 h, then heated at reflux for 2.5 h, after which time an additional amount of dimethylamine (2M in THF, 0.524 mL, 1.05 mmol) was added and the reaction mixture heated at reflux for 4 h. The reaction mixture was cooled, concentrated, and the residue dissolved in anhydrous DMF (0.75 mL), to which dimethylamine (2M in THF, 0.87 mL, 1.7 mmol) was added and the reaction mixture was stirred at rt for 18 h, then heated to 45 °C for 5 h, cooled to rt, concentrated, diluted with water, and extracted with CH_2Cl_2 (3 x). The organic phase was washed with brine (1 x), dried over $MgSO_4$, filtered and concentrated. Heptane was added and concentrated (2 x) to remove residual DMF. The residue was purified by silica column chromatography using a gradient of 6% to 12% MeOH in CH_2Cl_2 to afford 3-(6-chloropyridin-3-yl)-*N*,*N*-dimethylpropan-1-amine (**38**) as a very pale yellow oil (32 mg, 46%). ^{1}H NMR (500 MHz, $CDCl_3$) δ 8.23 (d, *J* = 2.3 Hz, 1H), 7.48 (dd, *J* = 8.2, 2.5 Hz, 1H), 7.24 (d, *J* = 8.1 Hz, 1H), 2.67 – 2.60 (m, 2H), 2.30 – 2.24 (m, 2H), 2.22 (s, 6H), 1.77 (m, 2H). LRMS (ESI^+) 199.10 $(M + H)^+$.

To a suspension of **38** (0.030 g, 0.15 mmol) and **32** (0.032 g, 0.15 mmol) in anhydrous toluene (1.5 mL) was added NaO^{t}Bu (0.022 g, 0.23 mmol), tris(dibenzylideneacetone)dipalladium(0) (0.003, 0.003 mmol), and Xantphos (0.005 g, 0.009 mmol). The flask was evacuated and backfilled with argon (3 x), and heated to 100 °C for 18h. The reaction mixture was cooled to rt, diluted with EtOAc, washed with water (1 x), brine (1 x), dried over Na_2SO_4, filtered, and concentrated. The residue was purified by silica column chromatography using a gradient of 5% to 18% MeOH in CH_2Cl_2 to afford the title compound **15** as a white solid (40 mg, 71%). ^{1}H NMR (500 MHz, $CDCl_3$) δ 8.79 (s, 1H), 8.72 (d, *J* = 1.0 Hz, 1H), 8.50 (br s, 1H), 8.27-8.23 (m, 2H), 7.88 (d, *J* = 7.6 Hz, 1H), 7.74 (br s, 1H), 7.55 (dd, *J* = 8.4, 2.4 Hz, 1H), 7.48 – 7.42 (m, 1H), 7.42 – 7.36 (m, 1H), 7.10 (br d, *J* = 8.3 Hz, 1H), 2.69 – 2.62 (m, 2H), 2.35 – 2.30 (m, 2H), 2.25 (s, 6H), 1.81 (m, 2H). ^{13}C NMR (126 MHz, $CDCl_3$) δ 160.61, 158.64, 156.60, 150.95, 147.68, 145.33, 141.38, 138.53, 132.22, 132.03, 124.81, 123.94, 121.09, 113.72, 112.80, 95.31, 58.97, 45.61, 30.12, 29.34. HRMS (ESI^+): calcd for $C_{21}H_{24}N_7$ $(M + H)^+$, 374.2088; found 374.2096.

(*S*)-6-(1*H*-Benzo[*d*]imidazol-1-yl)-*N*-(5-((1-methylpyrrolidin-2-yl)methoxy)pyridin-2-yl)pyrimidin-4-amine (**16**)

Diisopropyl azodicarboxylate (0.453 g, 0.441 mL, 2.24 mmol) was added drop-wise to a solution of 2-bromo-5-hydroxypyridine **28** (0.300 g, 1.72 mmol), (*S*)-(1)-1-methyl-2-pyrrolidinemethanol (0.199

g, 0.205 mL, 1.72 mmol), and triphenylphosphine (0.588 g, 2.24 mmol) in anhydrous THF (6 mL) at 0 °C. After the addition the reaction mixture was allowed to warm to rt, and stirred for 23 h, concentrated and the residue was dissolved in EtOAc, washed with 1 M NaOH (aq.) (1 x), brine (1 x), dried over $MgSO_4$, filtered and concentrated. The residue was purified by silica column chromatography using a gradient of 1.5 to 6% MeOH in CH_2Cl_2 to afford compound (*S*)-2-Bromo-5-((1-methylpyrrolidin-2-yl)methoxy)pyridine (**34b**) as a pale yellow oil (0.275 g, 59%). ^{1}H NMR (500 MHz, $CDCl_3$) δ 8.07 (d, *J* = 3.3 Hz, 1H), 7.35 (d, *J* = 8.7 Hz, 1H), 7.11 (dd, *J* = 8.7, 3.2 Hz, 1H), 3.97 (dd, *J* = 9.2, 5.2 Hz, 1H), 3.90 (dd, *J* = 9.2, 5.5 Hz, 1H), 3.13 - 3.08 (m, 1H), 2.68 - 2.63 (m, 1H), 2.46 (s, 3H), 2.35 – 2.27 (m, 1H), 2.07 - 1.98 (m, 1H), 1.88 – 1.75 (m, 2H), 1.75 - 1.67 (m, 1H). HRMS (ESI^+): calcd for $C_{11}H_{16}{}^{79}BrN_2O$ $(M + H)^+$, 271.0440; found 271.0442.

A flask was charged with **32** (58 mg, 0.28 mmol), **34b** (75 mg, 0.28 mmol), tris(dibenzylideneacetone)dipalladium(0) (15 mg, 0.017 mmol), Xantphos (19 mg, 0.033 mmol), and cesium carbonate$_3$ (0.135 g, 0.415 mmol) in anhydrous dioxane (2.0 mL), then evacuated and backfilled with argon (4 x), sealed, and heated to 100 °C for 24 h, cooled to rt, diluted with EtOAc, and washed with water (1 x). The aqueous phase was filtered, then extracted with EtOAc (2 x), and the combined organic phases were dried over Na_2SO_4, filtered and concentrated. The residue was purified by silica column chromatography using a gradient of 3.5 to 10% MeOH in CH_2Cl_2 to afford the title compound **16** as an off-white solid (14 mg, 13%). ^{1}H NMR (500 MHz, $CDCl_3$) δ 8.76 (s, 1H), 8.71 (d, *J* = 1.0 Hz, 1H), 8.28 (br s, 1H), 8.21 (d, *J* = 7.8 Hz, 1H), 8.11 (d, *J* = 2.9 Hz, 1H), 7.88 (dd, *J* = 7.5, 0.8 Hz, 1H), 7.64 (br s, 1H), 7.46 – 7.41 (m, 1H), 7.41 – 7.36 (m, 1H), 7.33 (dd, *J* = 8.9, 3.0 Hz, 1H), 7.17 (br d, *J* = 8.9 Hz, 1H), 4.04 (dd, *J* = 9.1, 5.2 Hz, 1H), 3.97 (dd, *J* = 9.1, 5.5 Hz, 1H), 3.15 (t, *J* = 7.7 Hz, 1H), 2.73-2.66 (m, 1H), 2.51 (s, 3H), 2.41 – 2.28 (m, 1H), 2.06 (m, 1H), 1.91-1.72 (m, 3H). ^{13}C NMR (126 MHz, $CDCl_3$) δ 160.65, 158.69, 156.50, 151.77, 146.31, 145.27, 141.36, 134.52, 131.99, 125.52, 124.79, 123.91, 121.07, 113.94, 113.64, 94.54, 71.87, 64.51, 57.91, 41.90, 28.74, 23.23. HRMS (ESI^+): calcd for $C_{22}H_{24}N_7O$ $(M + H)^+$, 402.2037; found 402.2042.

(*R*)-6-(1*H*-Benzo[*d*]imidazol-1-yl)-*N*-(5-((1-methylpyrrolidin-2-yl)methoxy)pyridin-2-yl)pyrimidin-4-amine (**17**)

Benzimidazole (2.50 g, 21.16 mmol) was added to 4,6-dichloropyrimidine (7.88 g, 52.91 mmol) and cesium carbonate (13.79 g, 42.32 mmol) in DMF (100 mL) at 20°C under nitrogen. The resulting suspension was stirred at 20 °C for 2 hours. The reaction mixture was filtered and the filtrate was concentrated *in vacuo*. The residue was dissolved in CH_2Cl_2 (100 mL) and washed with water (100 mL). The organic layer was passed through a phase separating cartridge and concentrated under reduced pressure to give a beige solid. The crude product was purified by flash silica chromatography, elution gradient 0 to 50% EtOAc in isohexane. Pure fractions were evaporated to dryness to afford 1-(6-chloropyrimidin-4-yl)-1*H*-benzo[*d*]imidazole (**41a**) as a pale yellow solid (3.88 g, 79%). ^{1}H NMR (400 MHz, DMSO-d_6) δ 9.20 (s, 1H), 9.08 (d, *J* = 0.88 Hz, 1H), 8.59 – 8.44 (m, 1H), 8.37 (d, *J* = 0.95 Hz, 1H), 7.71 – 7.9 (m, 1H), 7.56 – 7.20 (m, 2H). LRMS (ESI^+): 231 $(M + H)^+$.

Diisopropyl azodicarboxylate (1.47 mL, 7.47 mmol) was added drop-wise to (*R*)-(1-methylpyrrolidin-2-yl)methanol (0.662 g, 5.75 mmol), 6-bromopyridin-3-ol (1.00 g, 5.75 mmol) and triphenylphosphine (1.96 g, 7.47 mmol) in THF (20 mL) at 0°C over a period of 10 minutes under nitrogen. The resulting solution was stirred at 0 °C for 15 minutes, warmed to room temperature and stirred for 16 hours. The reaction mixture was loaded directly onto an SCX column and eluted with methanol followed by 7N NH_3 in methanol. Fractions containing the desired product were concentrated under reduced pressure to give an orange oil. The crude product was purified by flash silica chromatography, elution gradient 0 to 10% MeOH in CH_2Cl_2. Pure fractions were evaporated to dryness to afford (*R*)-2-bromo-5-((1-methylpyrrolidin-2-yl)methoxy)pyridine (**42**) as a pale yellow oil

(0.374 g, 24%). ^{1}H NMR (400 MHz, $CDCl_3$) δ 8.06 (d, *J* = 3.0 Hz, 1H), 7.35 (d, *J* = 8.6 Hz, 1H), 7.10 (dd, *J* = 3.2, 8.7 Hz, 1H), 3.98 (dd, *J* = 5.2, 9.2 Hz, 1H), 3.90 (dd, *J* = 5.5, 9.2 Hz, 1H), 3.10 (ddd, *J* = 2.2, 7.0, 9.1 Hz, 1H), 2.65 (m, 1H), 2.46 (s, 3H), 2.31 (td, *J* = 7.2, 9.4 Hz, 1H), 2.12 – 1.94 (m, 1H), 1.92 – 1.57 (m, 3H). LRMS (ESI^+): 273 $(M + H)^+$.

Palladium(II) acetate (30.6 mg, 0.14 mmol) was added to benzophenone imine (0.274 mL, 1.63 mmol), **42** (369 mg, 1.36 mmol), Xantphos (157 mg, 0.27 mmol) and cesium carbonate (887 mg, 2.72 mmol) in dioxane (10 mL) at 20 °C under nitrogen. The resulting suspension was stirred at 100 °C for 16 hours then cooled to room temperature. The reaction mixture was diluted with CH_2Cl_2 (100 mL) and the solids were filtered off. The filtrate was washed with water (100 mL), and the organic layer concentrated under reduced pressure. The crude product was purified by flash silica chromatography, elution gradient 0 to 10% MeOH in CH_2Cl_2. Pure fractions were evaporated to dryness to afford compound (*R*)-*N*-(diphenylmethylene)-5-((1-methylpyrrolidin-2-yl)methoxy)pyridin-2-amine (**43a**) as a yellow gum (288 mg, 57.0%). LRMS (ESI^+): 372 $(M + H)^+$.

2 M HCl (aqueous) (0.969 mL, 1.94 mmol) was added to a stirred solution of **43a** (288 mg, 0.78 mmol) in THF (5 mL) at 23ºC. The resulting solution was stirred at room temperature for 16 hours. The reaction mixture was diluted with MeOH and purified by ion exchange chromatography, using an SCX-2 column. The desired product was eluted from the column using 7M NH_3 in methanol and pure fractions were evaporated to dryness to afford compound (*R*)-5-((1-methylpyrrolidin-2-yl)methoxy)pyridin-2-amine (**44a**) as a brown oil which solidified on standing (159 mg, 99%). LRMS (ESI^+): 208 $(M + H)^+$.

Tris(dibenzylideneacetone)dipalladium(0) (31.6 mg, 0.03 mmol) was added to **44a** (143 mg, 0.69 mmol), **41a** (159 mg, 0.69 mmol), sodium *t*-butoxide (99 mg, 1.03 mmol) and Xantphos (59.8 mg, 0.10 mmol) in toluene (6 mL) at 20°C under nitrogen. The resulting suspension was stirred at 100 °C for 16 hours then cooled to room temperature. The reaction mixture was concentrated *in vacuo*. The residue was dissolved in CH_2Cl_2 (100 mL) and washed with water (100 mL). The organic layer was passed through a phase separating cartridge and concentrated under reduced pressure. The crude product was purified by flash silica chromatography, elution gradient 0 to 10% MeOH in CH_2Cl_2. Pure fractions were evaporated to dryness to afford a yellow gum. This was re-purified by preparative HPLC (Waters XBridge Prep C18 OBD column, 5μ, 19 mmx 100 mm), using decreasingly polar mixtures of water (containing 1% NH_3) and MeCN as eluents. Fractions containing the desired compound were evaporated to dryness to afford the title compound **17** as a pale yellow solid (39.0 mg, 14%). ^{1}H NMR (400 MHz, DMSO-d_6, 21°C) δ 10.40 (s, 1H), 8.94 (s, 1H), 8.73 (d, *J* = 0.7 Hz, 1H), 8.24 (d, *J* = 8.2 Hz, 1H), 8.14 (m, 2H), 7.81 (d, *J* = 7.9 Hz, 1H), 7.66 (d, *J* = 7.9 Hz, 1H), 7.53 - 7.44 (m, 2H), 7.42 - 7.34 (m, 1H), 4.04 (dd, *J* = 9.5, 5.2 Hz, 1H), 3.89 (m, 1H), 3.03 – 2.88 (m, 1H), 2.64 - 2.52 (m, 1H), 2.37 (s, 3H), 2.19 (m, 1H), 2.04 - 1.88 (m, 1H), 1.79 - 1.53 (m, 3H). ^{13}C NMR (176 MHz, DMSO-d_6, 27°C) δ 160.66, 158.25, 158.19, 155.05, 150.78, 146.57, 144.53, 141.54, 134.00, 131.39, 124.82, 124.29, 123.35, 120.19, 114.22, 113.55, 94.14, 71.60, 63.56, 56.97, 41.24, 28.36, 22.54. HRMS (ESI^+): calcd for $C_{22}H_{24}N_7O$ $(M + H)^+$, 402.2037; found 402.2037.

6-(1*H*-Benzo[*d*]imidazol-1-yl)-*N*-(5-(2-morpholinoethoxy)pyridin-2-yl)pyrimidin-4-amine (**18**)

A mixture of 2-bromo-5-hydroxypyridine **28** (0.200 g, 1.15 mmol), 4-(2-chloroethyl)morpholine hydrochloride (0.246 g, 1.32 mmol), and K_2CO_3 (0.477 g, 3.45 mmol) in anhydrous DMF (4.0 mL) was heated to 70 °C for 17 h, cooled to rt, diluted with water, extracted with EtOAc (3 x). The organic phase was washed with brine (1 x), dried over $MgSO_4$, filtered and concentrated. Heptane was added and concentrated (2 x) to remove residual DMF. The residue was purified by silica column chromatography using a gradient of 1% to 2.5% MeOH in CH_2Cl_2 to afford compound 4-(2-((6-bromopyridin-3-yl)oxy)ethyl)morpholine (**34c**) as a pale yellow solid (283 mg, 86%). ^{1}H NMR (500 MHz, $CDCl_3$) δ 8.07 (d, *J* = 3.1 Hz, 1H), 7.36 (d, *J* = 8.5 Hz, 1H), 7.11 (dd, *J* = 8.7, 3.2 Hz, 1H), 4.13

(t, J = 5.7 Hz, 2H), 3.76 – 3.68 (m, 4H), 2.80 (t, J = 5.6 Hz, 2H), 2.61 – 2.47 (m, 4H). HRMS (ESI$^+$): calcd for $C_{11}H_{16}{}^{79}BrN_2O_2$ (M + H)$^+$, 287.0390; found 287.0392.

A flask was charged with **32** (50 mg, 0.24 mmol), **34c** (68 mg, 0.24 mmol), tris(dibenzylideneacetone)dipalladium(0) (6 mg, 0.007 mmol), Xantphos (8 mg, 0.01 mmol), and cesium carbonate (0.116 g, 0.355 mmol) in anhydrous dioxane (2.0 mL), then evacuated and backfilled with argon (3 x), sealed, and heated to 100 °C for 21 h, cooled to rt and added additional tris(dibenzylideneacetone)dipalladium(0) (6 mg, 0.007 mmol), Xantphos (8 mg, 0.01 mmol), and cesium carbonate (0.116 g, 0.355 mmol). The flask was evacuated and backfilled with argon (3 x), sealed, and heated to 100 °C for 18 h, cooled to rt, diluted with EtOAc, washed with water (1 x), brine (1 x), dried over $MgSO_4$, filtered and concentrated. The residue was purified by silica column chromatography using a gradient of 1% to 6.5% MeOH in CH_2Cl_2 to afford compound **18** as an off-white solid (47 mg, 48%). ^{1}H NMR (500 MHz, $CDCl_3$) δ 8.76 (s, 1H), 8.71 (d, J = 0.8 Hz, 1H), 8.26 (br s, 1H), 8.21 (d, J = 8.0 Hz, 1H), 8.11 (d, J = 2.9 Hz, 1H), 7.88 (d, J = 7.6 Hz, 1H), 7.59 (br s, 1H), 7.47 – 7.42 (m, 1H), 7.39 (m, 1H), 7.32 (dd, J = 8.9, 3.0 Hz, 1H), 7.19 (br d, J = 8.9 Hz, 1H), 4.18 (t, J = 5.6 Hz, 2H), 3.78 – 3.72 (m, 4H), 2.83 (t, J = 5.6 Hz, 2H), 2.64 – 2.52 (m, 4H). ^{13}C NMR (126 MHz, $CDCl_3$) δ 160.60, 158.73, 156.57, 151.45, 146.49, 145.31, 141.34, 134.63, 132.01, 125.75, 124.79, 123.94, 121.11, 113.91, 113.66, 94.56, 67.06, 57.81, 54.30. HRMS (ESI$^+$): calcd for $C_{22}H_{24}N_7O_2$ (M + H)$^+$, 418.1986; found 418.1999.

6-(1*H*-Benzo[*d*]imidazol-1-yl)-*N*-(5-(2-(piperidin-1-yl)ethoxy)pyridin-2-yl)pyrimidin-4-amine (**19**)

A mixture of 2-bromo-5-hydroxypyridine **28** (0.300 g, 1.72 mmol), 1-(2-chloroethyl)piperidine hydrochloride (0.381 g, 2.07 mmol), and K_2CO_3 (0.834 g, 6.03 mmol) in anhydrous DMF (6.0 mL) was heated to 70 °C for 21 h, cooled to rt, diluted with water, extracted with EtOAc (3 x). The organic phase was washed with brine (1 x), dried over $MgSO_4$, filtered and concentrated. Heptane was added and concentrated (2 x) to remove residual DMF. The residue was purified by silica column chromatography using a gradient of 2 to 3% MeOH in CH_2Cl_2 to afford compound 2-bromo-5-(2-(piperidin-1-yl)ethoxy)pyridine (**34d**) as a pale yellow oil (0.390 g, 79%). ^{1}H NMR (500 MHz, $CDCl_3$) δ 8.07 (d, J = 3.1 Hz, 1H), 7.35 (d, J = 8.6 Hz, 1H), 7.11 (dd, J = 8.6, 3.1 Hz, 1H), 4.11 (t, J = 5.9 Hz, 2H), 2.76 (t, J = 5.9 Hz, 2H), 2.49 (m, 4H), 1.60 (m, 4H), 1.45 (m, 2H). HRMS (ESI$^+$): calcd for $C_{12}H_{18}{}^{79}BrN_2O$ (M + H)$^+$, 285.0597; found 285.0613.

A flask was charged with **32** (55 mg, 0.26 mmol), **34d** (74 mg, 0.26 mmol), tris(dibenzylideneacetone)dipalladium(0) (14 mg, 0.016 mmol), Xantphos (18 mg, 0.031 mmol), and cesium carbonate (0.169 g, 0.519 mmol) in anhydrous dioxane (2.0 mL), then evacuated and backfilled with argon (4 x), sealed, and heated to 100 °C for 23 h, cooled to rt and added additional tris(dibenzylideneacetone)dipalladium(0) (14 mg, 0.016 mmol), Xantphos (18 mg, 0.031 mmol), and cesium carbonate (0.169 g, 0.519 mmol). The flask was evacuated and backfilled with argon (3 x), sealed, and heated to 100 °C for 23 h, cooled to rt, diluted with EtOAc, washed with water (1 x). The aqueous phase was filtered, then extracted with EtOAc (2 x), and the combined organic phases were washed with brine (1 x), dried over Na_2SO_4, filtered and concentrated. The residue was purified by silica column chromatography using a gradient of 5% to 8.5% MeOH in CH_2Cl_2 to afford the title compound **19** as a pale yellow solid (50 mg, 46%). ^{1}H NMR (500 MHz, $CDCl_3$) δ 8.76 (s, 1H), 8.71 (d, J = 0.9 Hz, 1H), 8.27 (br s, 1H), 8.21 (d, J = 8.0 Hz, 1H), 8.11 (d, J = 3.0 Hz, 1H), 7.88 (d, J = 7.5 Hz, 1H), 7.63 (br s, 1H), 7.46 – 7.41 (m, 1H), 7.41-7.37 (m, 1H), 7.32 (dd, J = 8.9, 3.0 Hz, 1H), 7.17 (br d, J = 8.9 Hz, 1H), 4.17 (t, J = 5.9 Hz, 2H), 2.80 (t, J = 5.9 Hz, 2H), 2.53 (br s, 4H), 1.66-1.59 (m, 4H), 1.51 – 1.41 (m, 2H). ^{13}C NMR (126 MHz, $CDCl_3$) δ 160.66, 158.69, 156.51, 151.58, 146.35, 145.27, 141.35, 134.67, 131.99, 125.68, 124.78, 123.92, 121.07, 113.94, 113.64, 94.54, 67.22, 58.07, 55.29, 26.03, 24.28. HRMS (ESI$^+$): calcd for $C_{23}H_{26}N_7O$ (M + H)$^+$, 416.2193; found 416.2196.

6-(1*H*-Benzo[*d*]imidazol-1-yl)-*N*-(5-((1-methylpiperidin-4-yl)oxy)pyridin-2-yl)pyrimidin-4-amine (**20**)

Diisopropyl azodicarboxylate (0.302 g, 0.294 mL, 1.49 mmol) was added drop-wise to a solution of 2-bromo-5-hydroxypyridine **28** (0.200 g, 1.15 mmol), 1-methyl-4-piperidinol (0.172 g, 1.49 mmol), and triphenylphosphine (0.392 g, 1.49 mmol) in anhydrous THF (5 mL) at 0 °C. After the addition the reaction mixture was allowed to warm to rt, and stirred for 27.5 h, concentrated and the residue dissolved in EtOAc, washed with saturated $NaHCO_3$ (aq.) (1 x), brine (1 x), dried over $MgSO_4$, filtered and concentrated. The residue was purified by silica column chromatography using a gradient of 1% to 6.5% MeOH in CH_2Cl_2 to afford compound 2-bromo-5-((1-methylpiperidin-4-yl)oxy)pyridine (**34e**) as a white solid (164 mg, 53%). ^{1}H NMR (500 MHz, $CDCl_3$) δ 8.05 (d, *J* = 3.1 Hz, 1H), 7.35 (d, *J* = 8.8 Hz, 1H), 7.09 (dd, *J* = 8.7, 3.1 Hz, 1H), 4.31 (m, 1H), 2.31 (s, 3H), 2.71–2.64 (m, 2H), 2.32 - 2.26 (m, 2H), 2.05 – 1.95 (m, 2H), 1.88 – 1.80 (m, 2H). HRMS (ESI$^+$): calcd for $C_{11}H_{16}{}^{79}BrN_2O$ (M + H)$^+$, 271.0440; found 271.0443.

A flask was charged with **32** (50 mg, 0.24 mmol), **34e** (64 mg, 0.24 mmol), tris(dibenzylideneacetone)dipalladium(0) (6 mg, 0.007 mmol), Xantphos (8 mg, 0.01 mmol), and cesium carbonate(0.116 g, 0.355 mmol) in anhydrous dioxane (2.0 mL), then evacuated and backfilled with argon (3 x), sealed, and heated to 100 °C for 17 h, cooled to rt and added additional tris(dibenzylideneacetone)dipalladium(0) (13 mg, 0.014 mmol), Xantphos (16 mg, 0.028 mmol), and cesium carbonate (0.116 g, 0.355 mmol). The flask was evacuated and backfilled with argon (3 x), sealed, and heated to 100 °C for 7 h, cooled to rt, diluted with EtOAc, washed with water (1 x). The aqueous phase was extracted with EtOAc (2 x), and the combined organic phases were washed with brine (1 x), dried over $MgSO_4$, filtered and concentrated. The residue was purified by silica column chromatography using a gradient of 3% to 12% MeOH in CH_2Cl_2 to afford the title compound **20** as an off-white solid (51 mg, 54%). ^{1}H NMR (500 MHz, $CDCl_3$) δ 8.76 (s, 1H), 8.71 (d, *J* = 0.9 Hz, 1H), 8.27 (br s, 1H), 8.22 (d, *J* = 8.1 Hz, 1H), 8.11 (d, *J* = 2.9 Hz, 1H), 7.88 (d, *J* = 7.6 Hz, 1H), 7.65 (br s, 1H), 7.47 – 7.41 (m, 1H), 7.41 – 7.35 (m, 1H), 7.31 (dd, *J* = 8.9, 3.0 Hz, 1H), 7.19 (br d, *J* = 8.8 Hz, 1H), 4.35-4.29 (m, 1H), 2.77-2.70 (m, 2H), 2.38-2.28 (m, 5H), 2.09-2.02 (m, 2H), 1.95 – 1.80 (m, 2H). ^{13}C NMR (126 MHz, $CDCl_3$) δ 160.63, 158.72, 156.57, 150.06, 146.47, 145.29, 141.34, 136.67, 132.03, 127.22, 124.80, 123.93, 121.09, 113.96, 113.70, 94.59, 73.99 52.65, 46.24, 30.90. HRMS (ESI$^+$): calcd for $C_{22}H_{24}N_7O$ (M + H)$^+$, 402.2037; found 402.2039.

6-(1*H*-Benzo[*d*]imidazol-1-yl)-*N*-(5-(4-methylpiperazin-1-yl)pyridin-2-yl)pyrimidin-4-amine (**21**)

To a solution of 2-bromo-5-iodopyridine **33** (0.368 g, 1.30 mmol) and 1-methylpiperazine (0.100 g, 0.111 mL, 0.998 mmol) in anhydrous toluene (10.0 mL) was added NaO^{t}Bu (0.144 g, 1.50 mmol), tris(dibenzylideneacetone)dipalladium(0) (18 mg, 0.020 mmol), and Xantphos (35 mg, 0.060 mmol). The flask was evacuated and backfilled with argon (3 x), and the reaction mixture was stirred at rt for 17 h, then heated to 80 °C for 6 h, cooled to rt, diluted with EtOAc, washed with water (1 x), brine (1 x), dried over $MgSO_4$, filtered and concentrated. The residue was purified by silica column chromatography using a gradient of 2% to 4.5% MeOH in CH_2Cl_2 to afford compound 1-(6-bromopyridin-3-yl)-4-methylpiperazine (**34f**) as a dull yellow solid (180 mg, 70%). ^{1}H NMR (500 MHz, $CDCl_3$) δ 8.01 (d, *J* = 3.2 Hz, 1H), 7.30 (d, *J* = 8.8 Hz, 1H), 7.07 (dd, *J* = 8.8, 3.2 Hz, 1H), 3.24 – 3.18 (m, 4H), 2.59 – 2.54 (m, 4H), 2.35 (s, 3H). HRMS (ESI$^+$): calcd for $C_{10}H_{15}{}^{79}BrN_3$ (M + H)$^+$, 256.0444; found 256.0446.To a suspension of **34f** (70 mg, 0.27 mmol) and **32** (58 mg, 0.27 mmol) in anhydrous toluene (2.5 mL) was added NaO^{t}Bu (39 mg, 0.41 mmol), tris(dibenzylideneacetone)dipalladium(0) (5 mg, 0.005 mmol), and Xantphos (10 mg, 0.016 mmol). The flask was evacuated and backfilled with argon (3 x), then heated to 100 °C for 6 h, cooled to rt, diluted with EtOAc, washed with water (1 x). The aqueous phase was filtered, extracted with EtOAc

(1 x), and the combined organic phases were washed with brine (1 x), dried over Na_2SO_4, filtered and concentrated. The residue was purified by silica column chromatography using a gradient of 2.5% to 11% MeOH in CH_2Cl_2 to afford the title compound **21** as an off-white solid (65 mg, 62%). ^{1}H NMR (500 MHz, DMSO-d$_6$) δ 10.26 (s, 1H), 8.91 (s, 1H), 8.70 (d, *J* = 0.9 Hz, 1H), 8.23 (d, *J* = 8.2 Hz, 1H), 8.14 (br s, 1H), 8.08 (d, *J* = 3.1 Hz, 1H), 7.81 (d, *J* = 7.9 Hz, 1H), 7.59 (br d *J* = 8.5 Hz, 1H), 7.51 – 7.44 (m, 2H), 7.38 (m, 1H), 3.19 – 3.09 (m, 4H), 2.49 – 2.44 (m, 4H), 2.23 (s, 3H). ^{13}C NMR (126 MHz, DMSO-d$_6$) δ 160.70, 158.31, 155.05, 145.37, 144.53, 142.82, 141.62, 134.64, 131.45, 125.85, 124.35, 123.40, 120.24, 113.88, 113.59, 94.15, 54.40, 48.25, 45.72. HRMS (ESI$^+$): calcd for $C_{21}H_{23}N_8$ (M + H)$^+$, 387.2040; found 387.2032.

(*S*)-6-(1*H*-Benzo[*d*]imidazol-1-yl)-*N*-(5-(2,4-dimethylpiperazin-1-yl)pyridin-2-yl)pyrimidin-4-amine (**22**)

A flask was charged with (3*S*)-1,3-dimethylpiperazine dihydrochloride (150 mg, 0.800 mmol), 2-bromo-5-iodopyridine **33** (296 mg, 1.30 mmol, 1.3 eq.), sodium *t*-butoxide (270 mg, 2.80 mmol, 3.5 eq.), Xantphos (28 mg, 0.050 mmol, 0.06 eq.) and tris(dibenzylideneacetone)dipalladium(0) (15 mg, 0.016 mmol, 0.02 eq.) and anhydrous toluene (8 mL). The reaction mixture was degassed by vacuum/argon cycles (3 x) and heated to 100 °C for 32 h. The reaction mixture was diluted with EtOAc (30 ml) and washed with water (20 mL). Solids formed, therefore the aqueous layer was filtered and extracted with EtOAc (20 mL). The combined organic layer was washed with brine (30 mL), dried (Na_2SO_4) and concentrated *in vacuo*. The resulting residue was purified by silica column chromatography (0-10% MeOH/ CH_2Cl_2) to afford the product (*S*)-1-(6-bromopyridin-3-yl)-2,4-dimethylpiperazine (**34g**) as a brown oil (52 mg, 24%). ^{1}H NMR (500 MHz, $CDCl_3$) δ 8.01 (d, *J* = 3.1 Hz, 1H), 7.32 (d, *J* = 8.7 Hz, 1H), 7.10 (dd, *J* = 8.7, 3.0 Hz, 1H), 3.88 – 3.83 (m, 1H), 3.37 – 3.22 (m, 1H), 3.22 – 3.07 (m, 1H), 2.94 – 2.80 (m, 1H), 2.68 (dd, *J* = 11.2, 3.3 Hz, 1H), 2.59 (br d, *J* = 9.9 Hz, 1H), 2.48 – 2.42 (m, 1H), 2.41 (s, 3H), 1.13 (d, *J* = 6.5 Hz, 3H). ^{13}C NMR (126 MHz, $CDCl_3$): δ 145.17, 127.73, 126.99, 60.36, 54.87, 50.70, 45.93, 44.28, 13.80. HRMS (ESI$^+$): calcd for $C_{11}H_{17}{}^{79}BrN_3$ (M + H)$^+$, 270.0606; found 270.0609.

A flask was charged with **34g** (51 mg, 0.19 mmol), **32** (48 mg, 0.23 mmol, 1.2 eq.), sodium *t*-butoxide (27 mg, 0.28 mmol, 1.5 eq.), Xantphos (6.6 mg, 0.010 mmol, 0.06 eq.) and tris(dibenzylideneacetone)dipalladium(0) (3.5 mg, 0.0030 mmol, 0.02 eq.) and anhydrous toluene (2 mL). The reaction mixture was degassed by vacuum/argon cycles (3 x) and heated to 100 °C for 4.5 h. At this point LCMS showed a small amount of remaining starting material, therefore further Xantphos (6.6 mg, 0.010 mmol, 0.06 eq.) and tris(dibenzylideneacetone)dipalladium(0) (3.5 mg, 0.0030 mmol, 0.02 eq.) were added and the reaction mixture stirred at 100 °C for a further 12 h. The reaction mixture was diluted with CH_2Cl_2 (30 mL) and washed with water (20 mL). Solids formed therefore the aqueous layer was filtered and extracted with CH_2Cl_2 (20 mL). The combined organic layer was washed with brine (30 mL), dried (Na_2SO_4) and concentrated *in vacuo*. The resulting residue was purified by silica column chromatography (0-20% MeOH/ CH_2Cl_2) to afford the title compound **22** as a dark yellow solid (42 mg, 56%).^{1}H NMR (500 MHz, MeOD) δ 8.87 (s, 1H), 8.64 (s, 1H), 8.24 (d, *J* = 8.2 Hz, 1H), 8.16 (br s, 1H), 8.08 (t, *J* = 1.5, 1H), 7.77 (d, *J* = 8.0 Hz, 1H), 7.50–7.48 (m, 2H), 7.46 (td, *J* = 7.7, 1.1 Hz, 1H), 7.40 (td, *J* = 7.5, 1.1 Hz, 1H), 3.69 – 3.63 (m, 1H), 3.18 (ddd, *J* = 11.6, 6.4, 3.3 Hz, 1H), 3.11 (ddd, *J* = 11.9, 7.3, 3.2 Hz, 1H), 2.72 – 2.69 (m, 1H), 2.66 (dd, *J* = 11.7, 3.2 Hz, 1H), 2.53 (ddd, *J* = 10.8, 7.4, 3.1 Hz, 1H), 2.47 (dd, *J* = 11.2, 5.7 Hz, 1H), 2.35 (s, 3H), 1.04 (d, *J* = 6.4 Hz, 3H). ^{13}C NMR (126 MHz, MeOD) δ 160.6, 157.7, 155.1, 146.7, 143.4, 141.7, 140.9, 138.8, 131.0, 129.2, 124.0, 123.2, 119.0, 113.6, 113.0, 94.1, 60.5, 54.5, 51.4, 44.4, 13.2. HRMS (ESI$^+$): calcd for $C_{22}H_{24}N_8$ (M + H)$^+$, 401.2197; found: 401.2190.

6-(1*H*-Benzo[*d*]imidazol-1-yl)-*N*-(5-(4-(dimethylamino)piperidin-1-yl)pyridin-2-yl)pyrimidin-4-amine (**23**)

To a solution of 2-bromo-5-iodopyridine **33** (0.366 g, 1.29 mmol) and 4-dimethylaminopiperidine (0.127 g, 0.991 mmol) in anhydrous toluene (10 mL) was added NaO^{t}Bu (0.143 g, 1.49 mmol), Pd_2dba_3 (18 mg, 0.020 mmol), and Xantphos (34 mg, 0.059 mmol). The flask was evacuated and backfilled with argon (3 x), then heated to 100 °C for 6 h, cooled to rt, diluted with EtOAc, washed with water (1 x). The aqueous phase was filtered, extracted with EtOAc (1 x), and the combined organic phases were washed with brine (1 x). An emulsion in the aqueous phase was then extracted with EtOAc (1 x), and washed with brine (2 x). The combined organic phases were dried over $MgSO_4$, filtered and concentrated. The residue was purified by silica column chromatography using a gradient of 5% to 10% MeOH in CH_2Cl_2 to afford compound 1-(6-bromopyridin-3-yl)-*N*,*N*-dimethylpiperidin-4-amine (**34h**) as a pale yellow solid (155 mg, 55%). ^{1}H NMR (500 MHz, $CDCl_3$) δ 8.01 (d, *J* = 3.2 Hz, 1H), 7.28 (d, *J* = 8.8 Hz, 1H), 7.08 (dd, *J* = 8.8, 3.2 Hz, 1H), 3.68 (d, *J* = 12.7 Hz, 2H), 2.77 (td, *J* = 12.3, 2.6 Hz, 2H), 2.31 (s, 6H), 2.30 - 2.25 (m, 1H), 1.99 – 1.89 (m, 2H), 1.67 - 1.57 (m, 2H). HRMS (ESI$^+$): calcd for $C_{12}H_{19}{}^{79}BrN_3$ $(M + H)^+$, 284.0757; found 284.0763.

To a suspension of **34h** (70 mg, 0.25 mmol) and **32** (52 mg, 0.25 mmol) in anhydrous toluene (2.4 mL) was added NaO^{t}Bu (36 mg, 0.37 mmol), tris(dibenzylideneacetone)dipalladium(0) (5 mg, 0.005 mmol), and Xantphos (9 mg, 0.02 mmol). The flask was evacuated and backfilled with argon (3 x), and the reaction mixture was heated to 100 °C for 4 h, cooled to rt, diluted with EtOAc, washed with water (1 x). The aqueous phase was filtered, extracted with EtOAc (1 x), and the combined organic phases were washed with brine (1 x), dried over Na_2SO_4, filtered and concentrated. The residue was purified by silica column chromatography using a gradient of 5% to 25% MeOH in CH_2Cl_2, then to 20% 2 M NH_3/MeOH in CH_2Cl_2 to afford compound **23** as an off-white solid (65 mg, 64%). ^{1}H NMR (500 MHz, $CDCl_3$) δ 8.75 (s, 1H), 8.69 (d, *J* = 1.0 Hz, 1H), 8.26 (br s, 1H), 8.21 (d, *J* = 8.1 Hz, 1H), 8.09 (d, *J* = 2.9 Hz, 1H), 7.87 (d, *J* = 7.8 Hz, 1H), 7.65 (br s, 1H), 7.46 – 7.41 (m, 1H), 7.41 – 7.36 (m, 1H), 7.34 (dd, *J* = 8.9, 3.0 Hz, 1H), 7.12 (br d, *J* = 8.8 Hz, 1H), 3.68 (m, 2H), 2.83-2.76 (m, 2H), 2.35 (s, 6H), 2.35-2.28 (m, 1H), 2.02-1.96 (m, 2H), 1.74-1.65 (m, 2H). ^{13}C NMR (126 MHz, $CDCl_3$) δ 160.74, 158.72, 156.49, 145.30, 145.25, 143.83, 141.36, 136.32, 132.05, 127.27, 124.75, 123.86, 121.06, 113.71, 113.68, 94.45, 61.94, 49.51, 41.86, 28.36. HRMS (ESI$^+$): calcd for $C_{23}H_{27}N_8$ $(M + H)^+$, 415.2353; found 415.2365.

(*S*)-6-(1*H*-Imidazo[4,5-*b*]pyridin-1-yl)-*N*-(5-((1-methylpyrrolidin-2-yl)methoxy)pyridin-2-yl)pyrimidin-4-amine (**24**)

4-Azabenzimidazole (72 mg, 0.60 mmol) was added to a suspension of NaH (60% in mineral oil, 26 mg, 0.66 mmol) in anhydrous DMF (0.75 mL) at 0 °C. The reaction mixture was stirred for 30 min at 0 °C then at rt for a further 15 min before 4-amino-6-chloropyrimidine (**31**) (65 mg, 0.50 mmol) was added. The reaction mixture was then heated to 100 °C for 16 h, cooled to rt, diluted with water and the resulting precipitate isolated by filtration, and washed with water. The crude material was purified by silica column chromatography using a gradient of 5 to 15% MeOH in CH_2Cl_2 to afford first the regioisomeric 7-azabenzimidazole derivative (2 mg, 2%), followed by the desired 4-azabenzimidazole derivative 6-(1*H*-Imidazo[4,5-*b*]pyridin-1-yl)pyrimidin-4-amine (**45**) as a white solid (30 mg, 28%). A NOESY NMR confirmed this regioisomer *via* a correlation between H7 of the azabenzimidazole moiety and the H5 of the pyrimidine. ^{1}H NMR (500 MHz, DMSO-d_6) δ 9.16 (s, 1H), 8.59 (dd, *J* = 8.2, 1.6 Hz, 1H), 8.53 (dd, *J* = 4.7, 1.6 Hz, 1H), 8.45 (d, *J* = 0.9 Hz, 1H), 7.42 (dd, *J* = 8.2, 4.7 Hz, 1H), 7.25 (br s, 2H), 6.83 (d, *J* = 0.9 Hz, 1H).

To a suspension of **45** (47 mg, 0.22 mmol) and **34b** (60 mg, 0.22 mmol) in anhydrous toluene (1.3 mL) and anhydrous DMF (0.9 mL) was added NaO^{t}Bu (32 mg, 0.33 mmol), tris(dibenzylideneacetone)dipalladium(0) (8 mg, 0.009 mmol), and Xantphos (15 mg, 0.027 mmol).

The flask was evacuated and backfilled with argon (3 x) and then heated to 100 °C for 15 h, and then cooled to rt for 3 h. The reaction mixture was diluted with EtOAc, washed with water (1 x). The aqueous phase and accompanying emulsion/precipitate was extracted with EtOAc (2 x). The combined organic phases were washed with brine (1 x), dried over Na_2SO_4, filtered, concentrated, and then diluted with heptane and concentrated (2 x) to remove residual DMF. The residue was purified by silica column chromatography using a gradient of 10% to 16% MeOH in CH_2Cl_2, and then switching to 7.5% 2 M NH_3/MeOH in CH_2Cl_2 to 10% to afford compound **24** as an off-white solid (9 mg, 10%). ^{1}H NMR (500 MHz, $CDCl_3$) δ 8.98 (s, 1H), 8.71 (s, 1H), 8.66 (dd, *J* = 4.7, 1.5 Hz, 1H), 8.63 (dd, *J* = 8.2, 1.5 Hz, 1H), 8.33 (br s, 1H), 8.12 (d, *J* = 2.9 Hz, 1H), 7.65 (br s, 1H), 7.38 (dd, *J* = 8.2, 4.7 Hz, 1H), 7.33 (dd, *J* = 8.9, 3.0 Hz, 1H), 7.14 (br d, *J* = 8.7 Hz, 1H), 4.08-4.00 (m, 1H), 3.97 (dd, *J* = 9.1, 5.5 Hz, 1H), 3.17-3.13 (m, 1H), 2.71-2.67 (m, 1H), 2.51 (s, 3H), 2.36-2.30 (m, 1H), 2.11 – 2.00 (m, 1H), 1.91 – 1.71 (m, 3H). HRMS (ESI$^+$): calcd for $C_{21}H_{22}N_8O$ $(M + H)^+$, 403.1989; found 403.1993.

6-(1*H*-Imidazo[4,5-*b*]pyridin-1-yl)-*N*-(5-(2-(piperidin-1-yl)ethoxy)pyridin-2-yl)pyrimidin-4-amine (**25**)

To a mixture of 4-amino-6-chloropyrimidine (**31**) (67 mg, 0.52 mmol) and **34d** (0.148 g, 0.519 mmol) in anhydrous toluene (5.2 mL) was added NaO^{t}Bu (75 mg, 0.78 mmol), tris(dibenzylideneacetone)dipalladium(0) (10 mg, 0.010 mmol), and Xantphos (18 mg, 0.031 mmol). The flask was evacuated and backfilled with argon (3 x) and then heated to 100 °C for 16.5 h, cooled to rt, diluted with EtOAc and water, filtered, and the aqueous phase was extracted with EtOAc (1 x). The combined organic phases were washed with brine (1x), dried over $MgSO_4$, filtered and concentrated. The residue was purified by silica column chromatography using a gradient of 5 to 12% MeOH in CH_2Cl_2, to afford 6-Chloro-*N*-(5-(2-(piperidin-1-yl)ethoxy)pyridin-2-yl)pyrimidin-4-amine (**46**) as a pale orange oil (52 mg, 30%). ^{1}H NMR (500 MHz, $CDCl_3$) δ 8.52 (d, *J* = 0.7 Hz, 1H), 8.05 (d, *J* = 3.4 Hz, 1H), 7.65 (br s, 1H), 7.47 (br s, 1H), 7.35 – 7.27 (m, 2H), 4.16 (t, *J* = 5.9 Hz, 2H), 2.80 (t, *J* = 5.9 Hz, 2H), 2.53 (br s, 4H), 1.67 – 1.58 (m, 4H), 1.47 (m, 2H). HRMS (ESI$^+$): calcd for $C_{16}H_{21}{}^{35}ClN_5O$ $(M + H)^+$, 334.1429; found 334.1432.

To a suspension of NaH (60% in mineral oil, 8 mg, 0.2 mmol) in anhydrous DMF (0.3 mL) at 0 °C was added 4-azabenzimidazole (21 mg, 0.17 mmol). The reaction mixture was stirred at 0 °C for 15 min, then warmed to rt for 20-25 min before a solution of **46** (48 mg, 0.14 mmol) in anhydrous DMF (0.3 mL) was added, and the reaction mixture was heated to 100 °C for 17 h, cooled to rt, diluted with water and stirred at rt for 4 h, after which time the precipitate was isolated by filtration and washed with water. The residue was purified by silica column chromatography using a gradient of 8 to 13% MeOH in CH_2Cl_2, and then switching to a gradient of 7.5 to 10% 2M NH_3/MeOH in CH_2Cl_2 to afford first the regioisomeric 7-azabenzimidazole derivative (7 mg, 12%), followed by the desired compound **25** as an off-white solid (26 mg, 43%). ^{1}H NMR (500 MHz, DMSO-d_6) δ 10.38 (s, 1H), 9.21 (s, 1H), 8.74 (d, *J* = 0.9 Hz, 1H), 8.62 (dd, *J* = 8.2, 1.5 Hz, 1H), 8.56 (dd, *J* = 4.7, 1.5 Hz, 1H), 8.16-8.13 (m, 2H), 7.64 (br d, *J* = 8.6 Hz, 1H), 7.52-7.47 (m, 2H), 4.14 (t, *J* = 5.9 Hz, 2H), 2.66 (t, *J* = 5.9 Hz, 2H), 2.47-2.42 (m, 4H), 1.53-1.47 (m, 4H), 1.42 – 1.33 (m, 2H). ^{13}C NMR (126 MHz, DMSO-d_6) δ 160.74, 158.32, 156.72, 154.88, 150.74, 146.47, 145.38, 143.74, 134.21, 125.02, 124.16, 122.29, 119.60, 114.32, 94.06, 66.49, 57.35, 54.35, 25.54, 23.88. HRMS (ESI$^+$): calcd for $C_{22}H_{25}N_8O$ $(M + H)^+$, 417.2146; found 417.2163.

References

1. P. Skehan, R. Storeng, D. Scudiero, A. Monks, J. Mcmahon, D. Vistica, J. T. Warren, H. Bokesch, S. Kenney and M. R. Boyd, *J. Natl. Cancer Inst.*, 1990, **82**, 1107-1112.
2. A. M. Lawrie, M. E. M. Noble, P. Tunnah, N. R. Brown, L. N. Johnson and J. A. Endicott, *Nat. Struct. Biol.*, 1997, **4**, 796-801.
3. M. Legraverend, P. Tunnah, M. Noble, P. Ducrot, O. Ludwig, D. S. Grierson, M. Leost, L. Meijer and J. Endicott, *J. Med. Chem.*, 2000, **43**, 1282-1292.
4. T. Wang, M. A. Block, S. Cowen, A. M. Davies, E. Devereaux, L. Gingipalli, J. Johannes, N. A. Larsen, Q. B. Su, J. A. Tucker, D. Whitston, J. Q. Wu, H. J. Zhang, M. Zinda and C. Chuaqui, *Bioorg. Med. Chem. Lett.*, 2012, **22**, 2063-2069.
5. W. Kabsch, *Acta Crystallogr., Sect. D: Biol. Crystallogr.*, 2010, **66**, 125-132.
6. P. R. Evans, *Acta Crystallogr., Sect. D: Biol. Crystallogr.* 2011, **67**, 282-292.
7. C. Vonrhein, C. Flensburg, P. Keller, A. Sharff, O. Smart, W. Paciorek, T. Womack and G. Bricogne, *Acta Crystallogr., Sect. D: Biol. Crystallogr.*, 2011, **67**, 293-302.
8. S. Bailey, *Acta Crystallogr., Sect. D: Biol. Crystallogr.*, 1994, **50**, 760-763.
9. S. Wlodek, A. G. Skillman and A. Nicholls, *Acta Crystallogr., Sect. D: Biol. Crystallogr.*, 2006, **62**, 741-749.
10. G. N. Murshudov, P. Skubak, A. A. Lebedev, N. S. Pannu, R. A. Steiner, R. A. Nicholls, M. D. Winn, F. Long and A. A. Vagin, *Acta Crystallogr., Sect. D: Biol. Crystallogr.*, 2011, **67**, 355-367.
11. P. Emsley, B. Lohkamp, W. G. Scott and K. Cowtan, *Acta Crystallogr., Sect. D: Biol. Crystallogr.*, 2010, **66**, 486-501.
12. Crystallographic statistics for the CDK2-compound **4** complex are as follows: space group $P2_12_12_1$, unit cell 53.4, 71.9, 72.3Å, resolution 1.83Å, 23,250 reflections from 10 3538 observations give 92.2% completeness with R_{merge} of 4.8% and mean I/□(I) of 17.1. The final model containing 2224 protein, 216 water, and 29 compound atoms has an R-factor of 17.3% (R_{free} using 5% of the data 20.4%). Mean temperature factors for the protein and the ligand are 26.4 and 39.3Å^2 respectively.
13. *The PyMOL Molecular Graphics System, Schrödinger, LLC.*
14. R. A. Laskowski, M. W. Macarthur, D. S. Moss and J. M. Thornton, *J. Appl. Crystallogr.*, 1993, **26**, 283-291.
15. M. W. Martin, J. Newcomb, J. J. Nunes, D. C. McGowan, D. M. Armistead, C. Boucher, J. L. Buchanan, W. Buckner, L. Chai, D. Elbaum, L. F. Epstein, T. Faust, S. Flynn, P. Gallant, A. Gore, Y. Gu, F. Hsieh, X. Huang, J. H. Lee, D. Metz, S. Middleton, D. Mohn, K. Morgenstern, M. J. Morrison, P. M. Novak, A. Oliveira-Dos-Santos, D. Powers, P. Rose, S. Schneider, S. Sell, Y. Tudor, S. M. Turci, A. A. Welcher, R. D. White, D. Zack, H. L. Zhao, L. Zhu, X. T. Zhu, C. Ghiron, P. Amouzegh, M. Ermann, J. Jenkins, D. Johnston, S. Napier and E. Power, *J. Med. Chem.*, 2006, **49**, 4981-4991.
16. D. Samson and E. Daltrozzo, *Helv. Chim. Acta*, 2011, **94**, 46-60.
17. Alper, P.; Azimioara, M.; Cow, C.; Epple, R.; Jiang, S.; Lelais, G.; Michellys, P.-Y.; Nguyen, T. N; Westscott-Baker, L.; Wu, B. WO2008097428A2, 2008.
